# Supplementary material for: Characterising the phenotypic evolution of circulating tumour cells during treatment
Source: Nat Commun. 2018 Apr 16;9:1482. doi: 10.1038/s41467-018-03725-8 (PMC5902511; doi:10.1038/s41467-018-03725-8)
Supplement: Supplementary file 1 — Supplementary Information(PDF 4855 kb) [file 41467_2018_3725_MOESM1_ESM.pdf]

Supplementary Information

Characterising the Phenotypic Evolution of Circulating Tumour Cells during Treatment

Tsao et al.

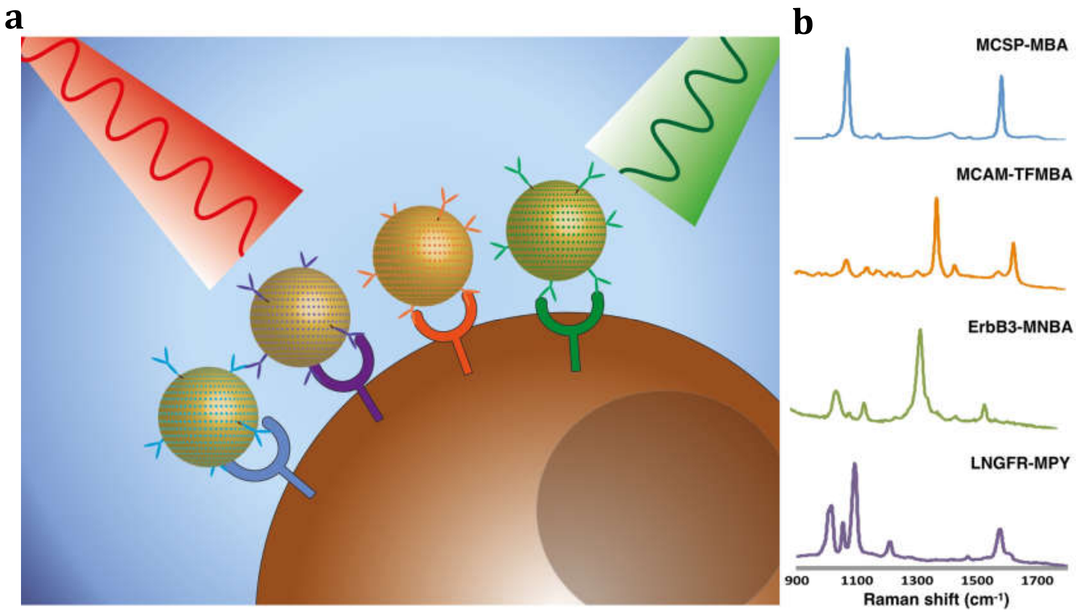

Supplementary Figure 1: Schematic illustration of (a) antibody-conjugated Raman reporter-coated gold nanoparticles (Ab-SERS labels) binding to cell surface markers and (b) the corresponding Raman fingerprints.

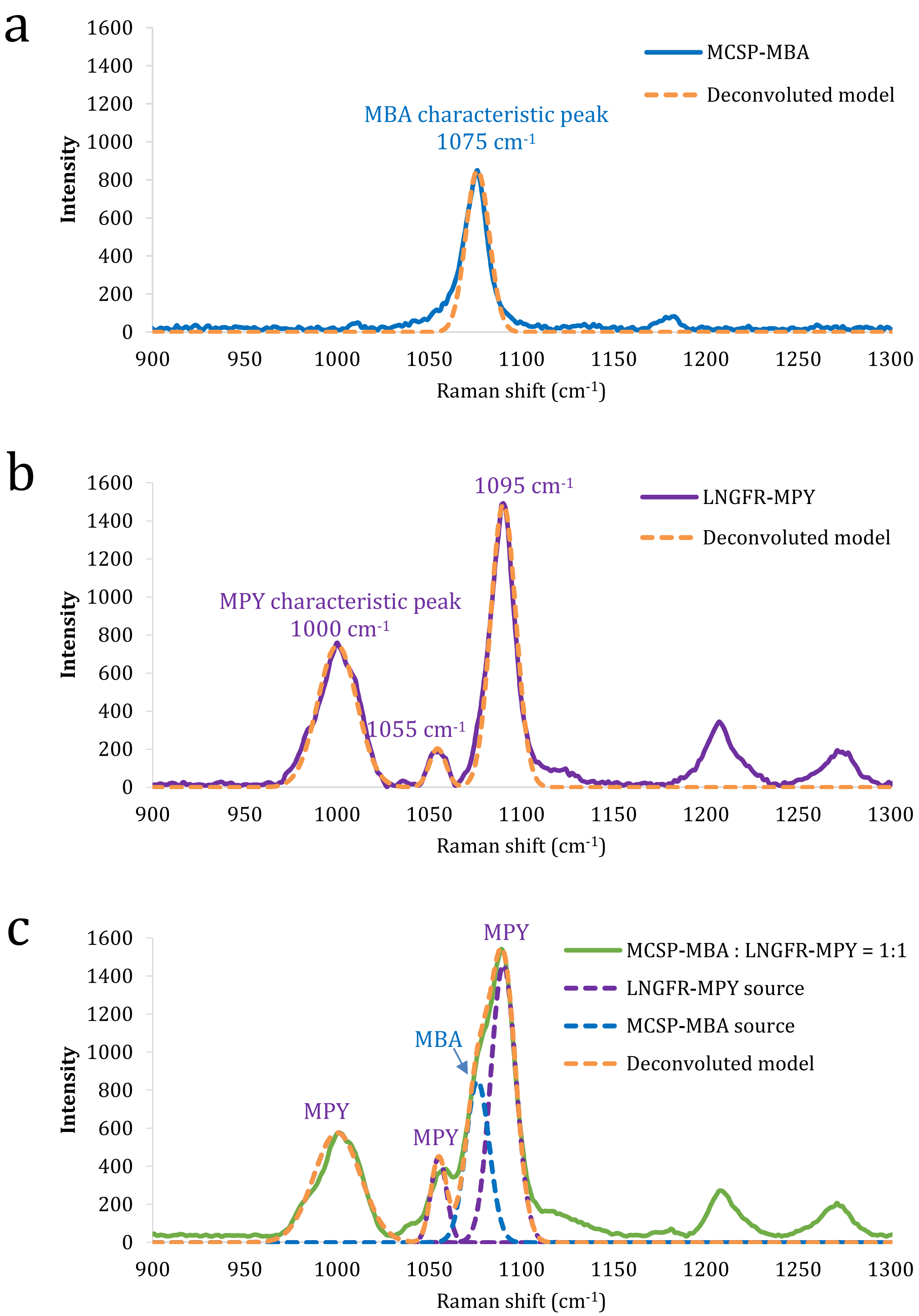
a
MCSP-MBA
Deconvoluted model
MBA characteristic peak
1075 cm-1
Intensity
Raman shift (cm-1)
b
LNGFR-MPY
Deconvoluted model
1095 cm-1
MPY characteristic peak
1000 cm-1
1055 cm-1
Intensity
Raman shift (cm-1)
c
MCSP-MBA : LNGFR-MPY = 1:1
LNGFR-MPY source
MCSP-MBA source
Deconvoluted model
MPY
MBA
MPY
MPY
Intensity
Raman shift (cm-1)

d

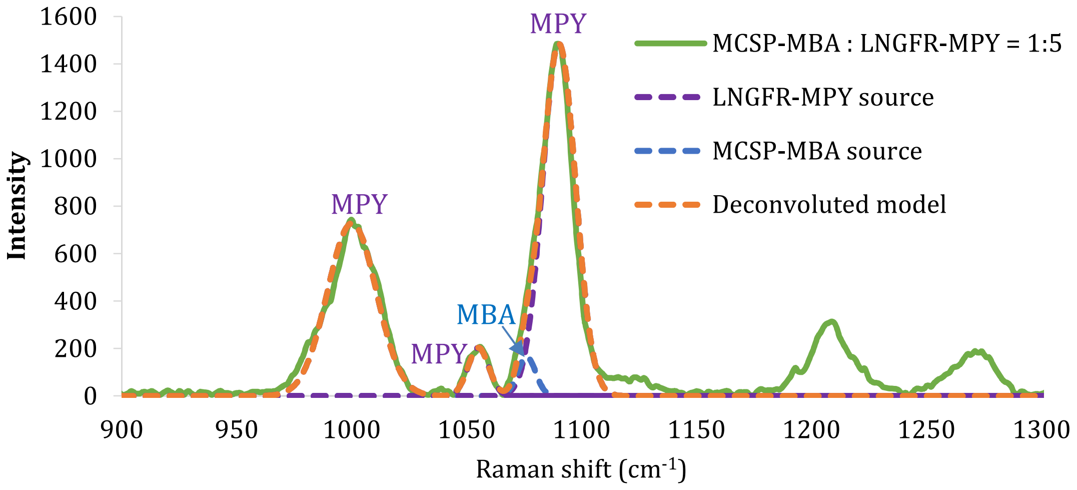

Supplementary Figure 2: SERS spectra of (a) MCSP-MBA-AuNPs, (b) LNGFR-MPY-AuNPs, (c) the mixture of MCSP-MBA- and LNGFR-MPY-AuNPs in a volume ratio of 1:1; and (d) the mixture of MCSP-MBA- and LNGFR-MPY-AuNPs in a volume ratio of 1:5. Solid lines are experimental spectra, and dash lines are deconvoluted models.

Supplementary Figure 2a, b shows that SERS spectra of individual MCSP-MBA and LNGFR-MPY match with their respective deconvoluted models, having only 0% and 1.8% variations for peak intensities at 1075 cm^{-1} and 1000 cm^{-1}, respectively (**Supplementary Table 1**). **Supplementary Fig. 2c, d** indicates the successful deconvolution of MCSP-MBA from LNGFR-MPY in the mixture of MCSP-MBA and LNGFR-MPY, mixed in volume ratios of 1:1 and 1:5, respectively, in which peaks belonging to MCSP-MBA and LNGFR-MPY can be clearly distinguished from each other. The corresponding peak intensities of MCSP-MBA source in both sets of the mixture, calculated from deconvoluted models, are in good agreement with respective peak intensities at individual MCSP-MBA levels (**Supplementary Table 1**), thus demonstrating deconvolution is capable of resolving the spectral overlap issue.

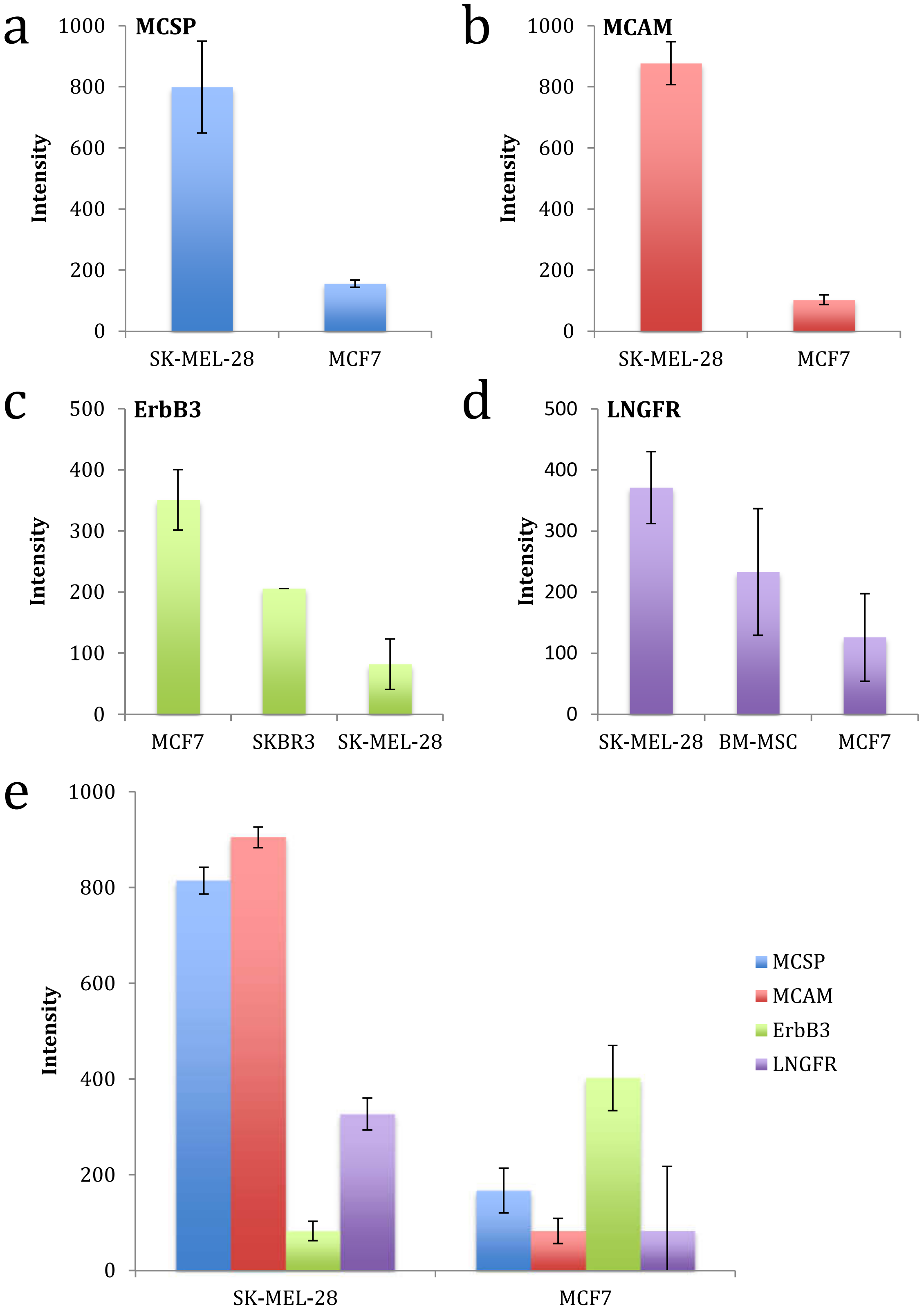
a
MCSP
Intensity
1000
800
600
400
200
0
SK-MEL-28
MCF7
b
MCAM
Intensity
1000
800
600
400
200
0
SK-MEL-28
MCF7
c
ErbB3
Intensity
500
400
300
200
100
0
MCF7
SKBR3
SK-MEL-28
d
LNGFR
Intensity
500
400
300
200
100
0
SK-MEL-28
BM-MSC
MCF7
e
Intensity
1000
800
600
400
200
0
SK-MEL-28
MCF7
MCSP
MCAM
ErbB3
LNGFR

f

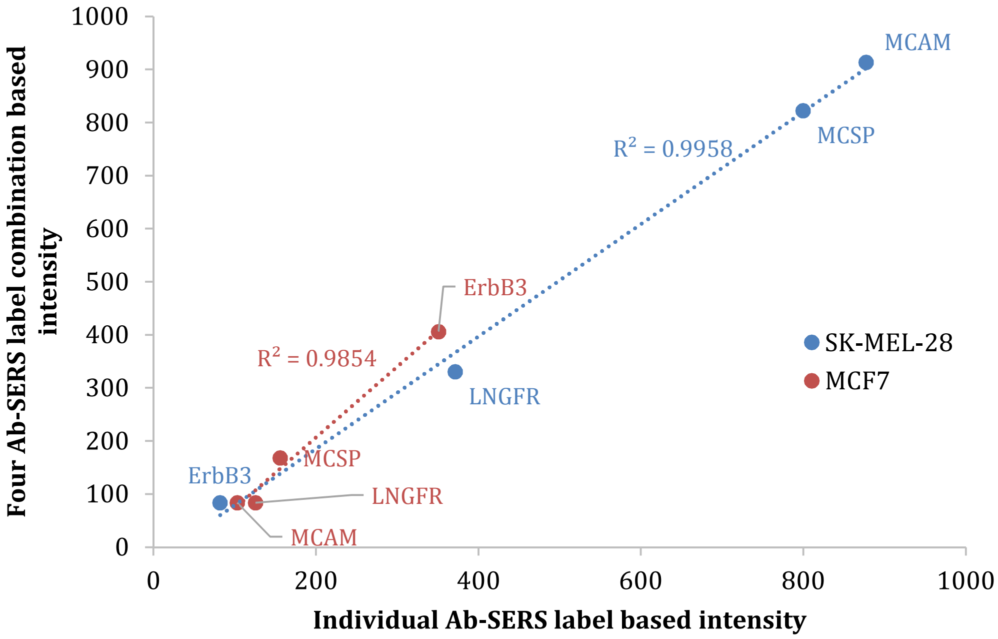

Supplementary Figure 3: Assay specificity studies of each Ab-SERS label alone and in combination in detecting reported cell lines.

(a) MCSP expression in SK-MEL-28 and MCF7 cell lines; (b) MCAM in SK-MEL-28 and MCF7 cell lines; (c) ErbB3 in MCF7, SKBR3, and SK-MEL-28 cell lines; (d) LNGFR in SK-MEL-28, BM-MSC and MCF7 cell lines; (e) four target combination in SK-MEL-28 and MCF7 cell lines. (f) Comparison between each antibody-SERS label alone and in combination for profiling four cell surface markers in two cell lines. Error bars represent s.d. with n = 3 (biological replicates).

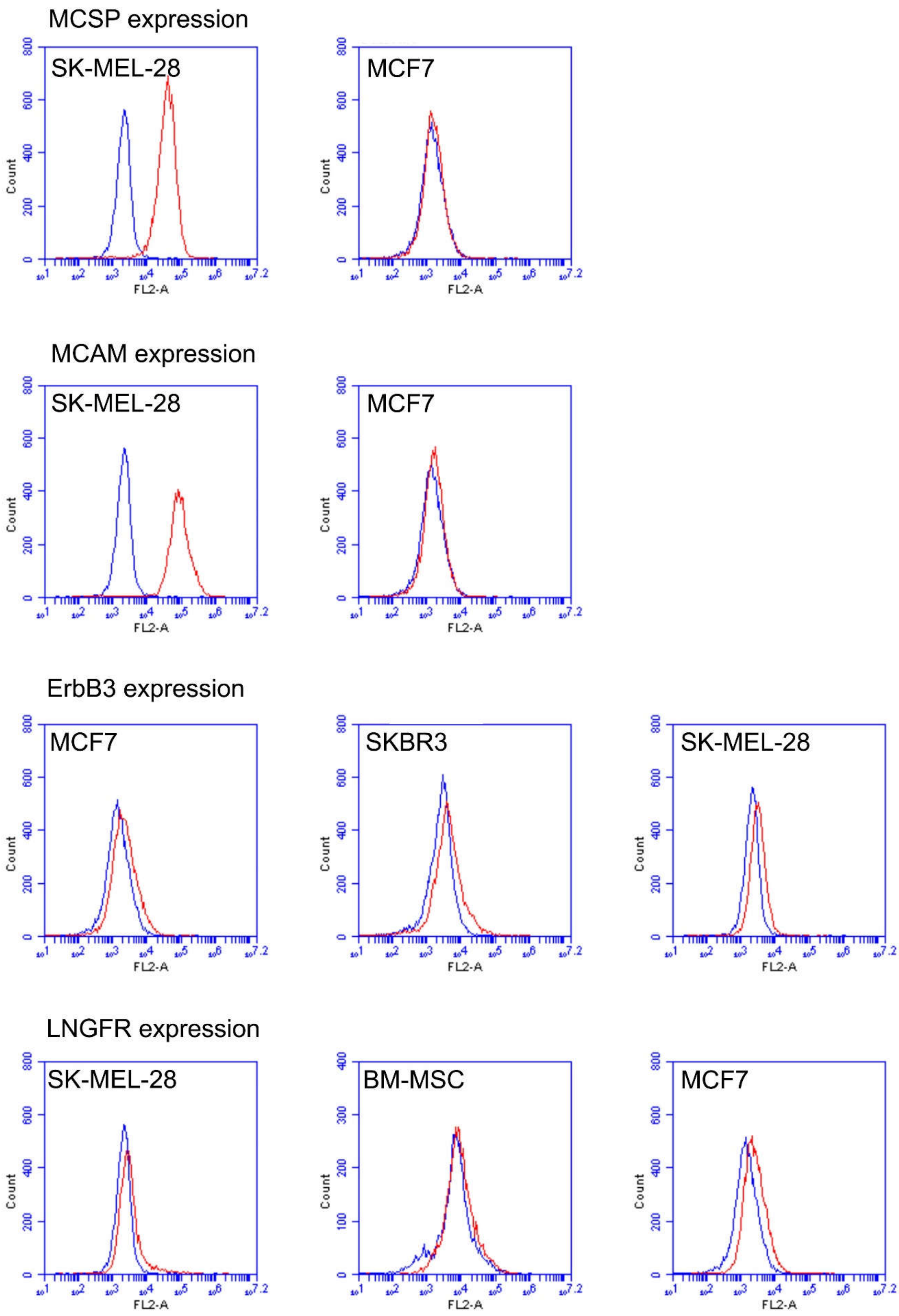

Supplementary Figure 4: Flow cytometry results for the different marker expression in different cell lines.

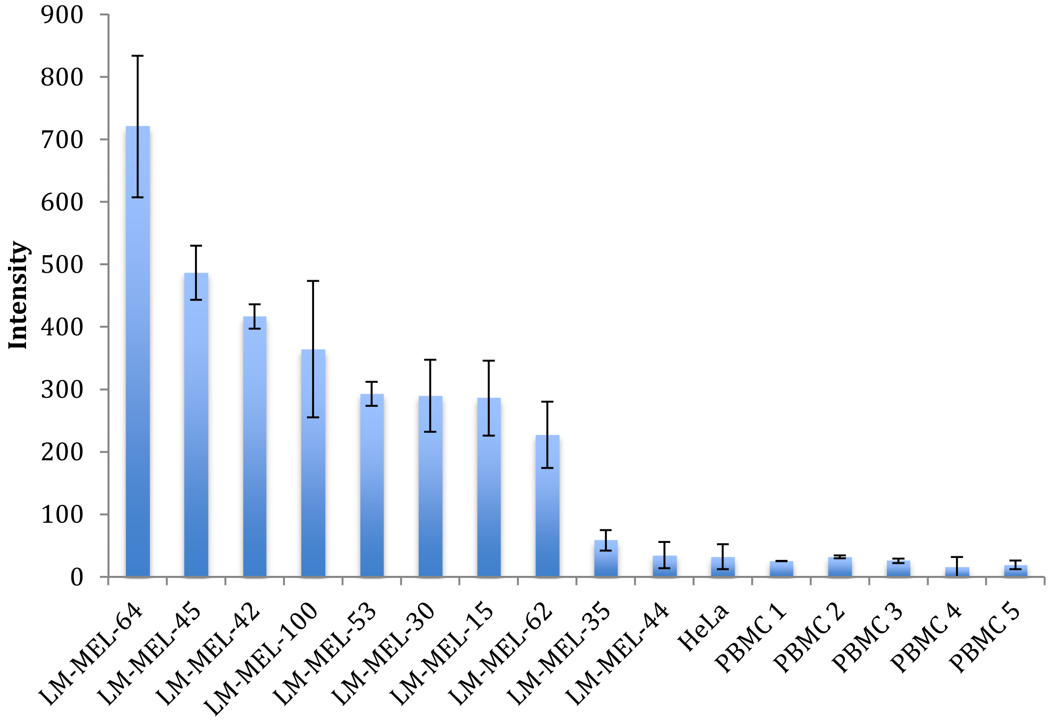

Supplementary Figure 5: Average Raman intensities of cell lines for MCSP expression.

Graph shows MCSP expression levels in 10 melanoma cell lines, HeLa and 5 healthy donor PBMC samples (10^7 cells). LM-MEL-44, HeLa, and PBMCs are negative for MCSP, and their intensities constitute background signals. Error bars represent s.d. with n = 3 (biological replicates).

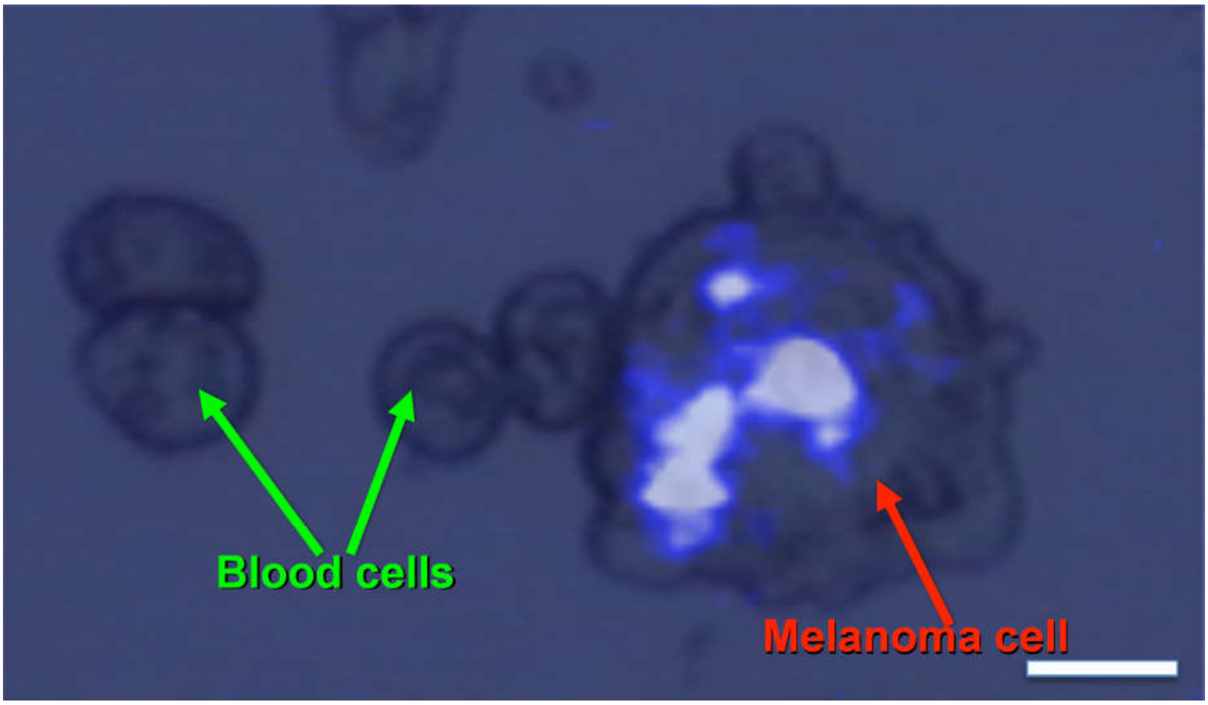

Supplementary Figure 6: SERS image of a melanoma cell spiked into blood cells.

The image shows the signal distribution of MCSP-MBA-AuNPs bound to MCSP on the melanoma cell surface while no signal was detected from blood cells. Scale bar, 10 µm.

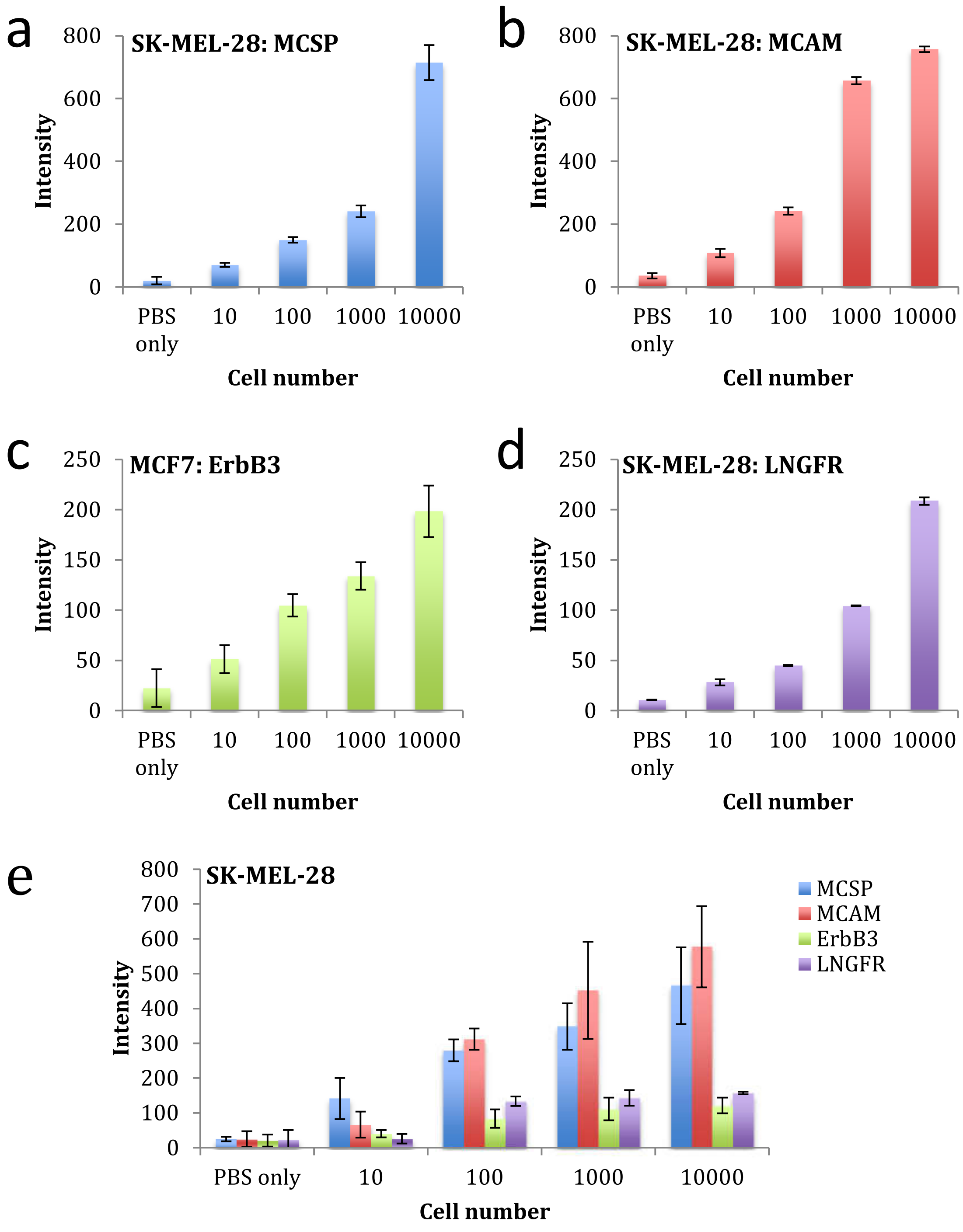

Supplementary Figure 7: Assay sensitivity study using different numbers of cells (conjugated with each Ab-SERS label alone and in combination) spiked into 1 mL of PBS.

(a) SK-MEL-28 labelled with MCSP-MBA-AuNPs; (b) SK-MEL-28 labelled with MCAM-TFMBA-AuNPs; (c) MCF7 labelled with ErbB3-MNBA-AuNPs; (d) SK-MEL-28 labelled with LNGFR-MPY-AuNPs; (e) SK-MEL-28 labelled with four target combination-SERS labels. Error bars represent s.d. with n = 3 (technical replicates).

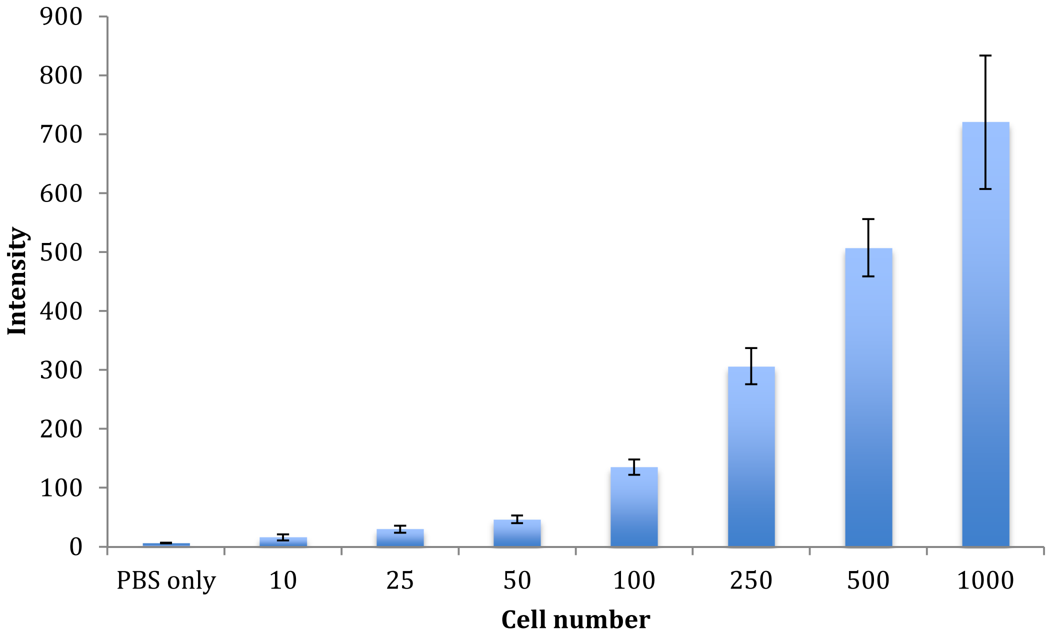

Supplementary Figure 8: Raman signals for different numbers of melanoma cells (LM-MEL-64 conjugated with MCSP-MBA-AuNPs) spiked into 1 mL of PBS.

This graph shows that the average Raman signals increase with increasing cell numbers. Raman spectroscopy can detect the presence of down to 10 cells depending on the expression level of the observed marker. Error bars represent s.d. with n = 3 (technical replicates).

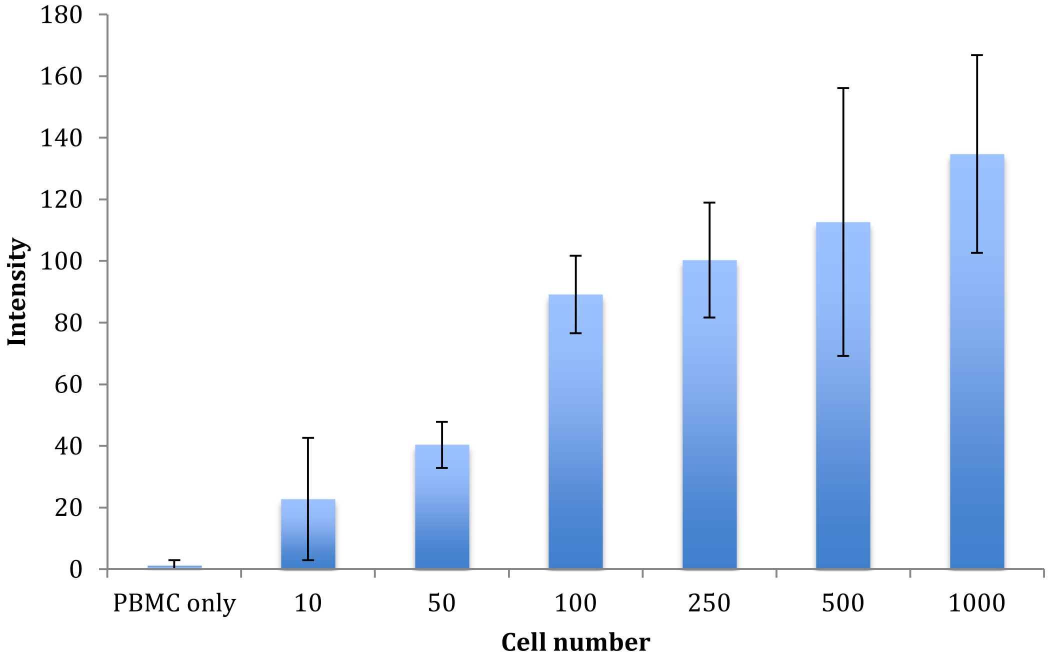

Supplementary Figure 9: Raman signals for different numbers of melanoma cells (LM-MEL-64 conjugated with MCSP-MBA-AuNPs) spiked into 10 mL of blood.

This graph shows that Raman spectroscopy can sensitively detect the presence of 10 cells. The large error bar is the result of unpredictable cell loss during density separation and CD45 depletion. Error bars represent s.d. with n = 3 (technical replicates).

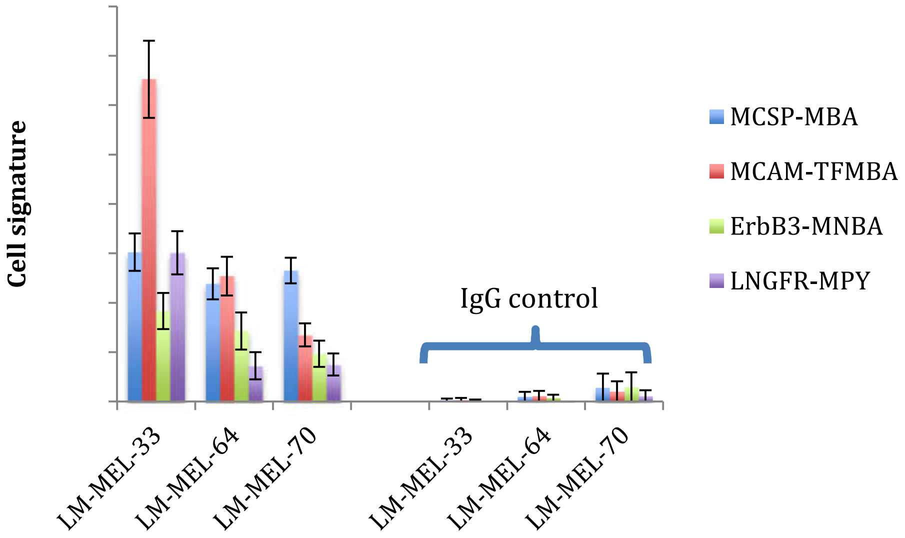

Supplementary Figure 10: Cell signatures collected by incubating LM-MEL-33, 64, and 70 cell line cells with MCSP/MCAM/ErbB3/LNGFR-SERS labels, or IgG-SERS labels (control). Error bars represent s.d. with n = 3 (biological replicates).

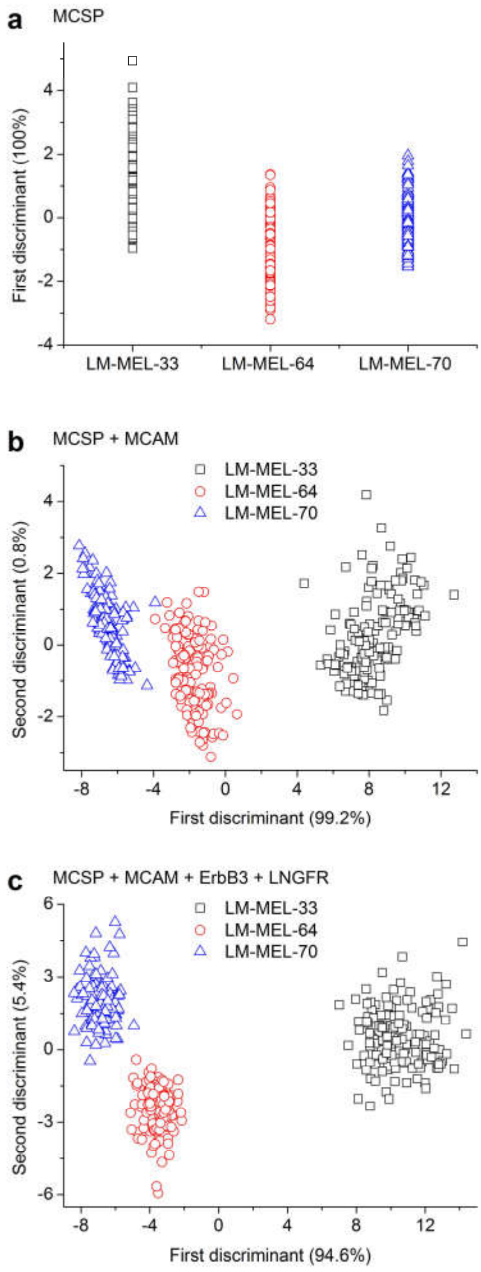

Supplementary Figure 11: Clustering of melanoma cell lines (LM-MEL-33, 64 and 70) after application of linear discriminant analysis (LDA) on SERS signals. (a) LDA based on only MCSP marker; (b) LDA based on MCSP and MCAM markers; (c) LDA based on four markers (MCSP, MCAM, ErbB3, and LNGFR).

Supplementary Figure 11a indicates one marker (i.e., MCSP) is insufficient to discriminate three melanoma cell lines. Using two markers significantly improves the discrimination accuracy (**Supplementary Fig. 11b**) and four markers completely cluster cell populations (**Supplementary Fig. 11c**).

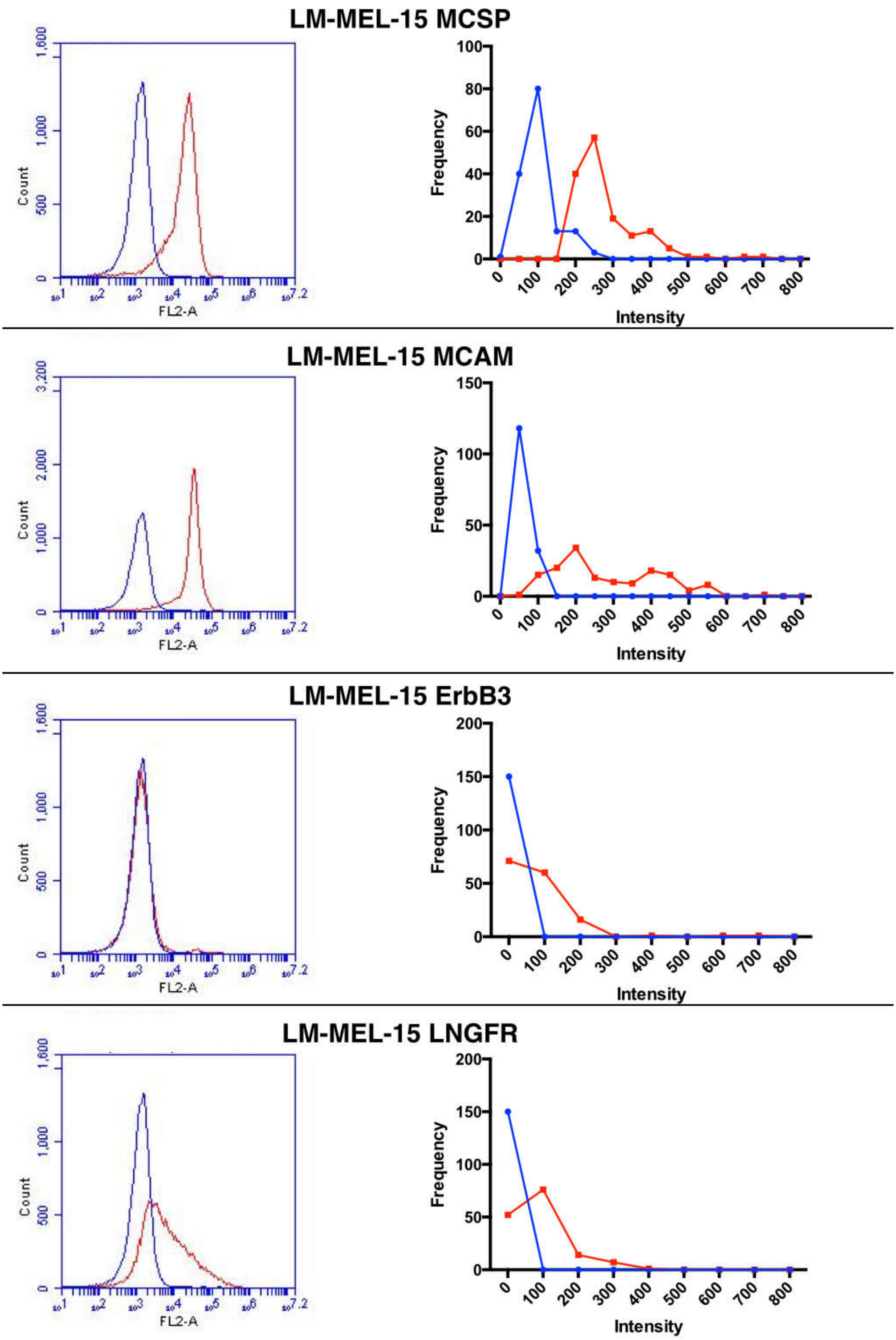

Supplementary Figure 12: Flow cytometry and Raman signal distribution of cell surface markers for LM-MEL-15 cell line.

Comparison of surface marker (MCSP, MCAM, ErbB3, and LNGFR) expression between Raman and flow cytometry. The proposed approach is effective in separating positively labelled cells from isotype-matched controls.

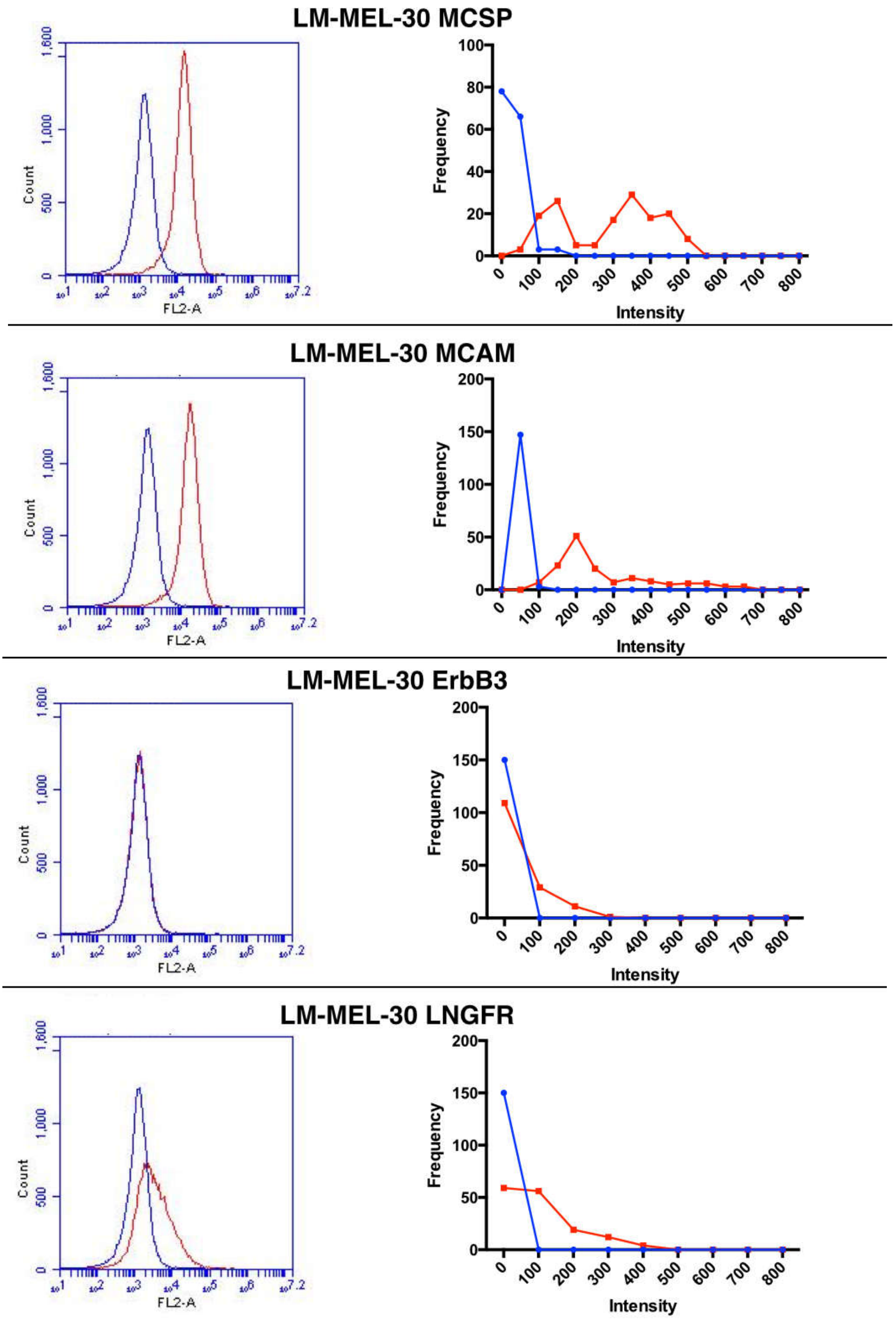

Supplementary Figure 13: Flow cytometry and Raman signal distribution of cell surface markers for LM-MEL-30 cell line.

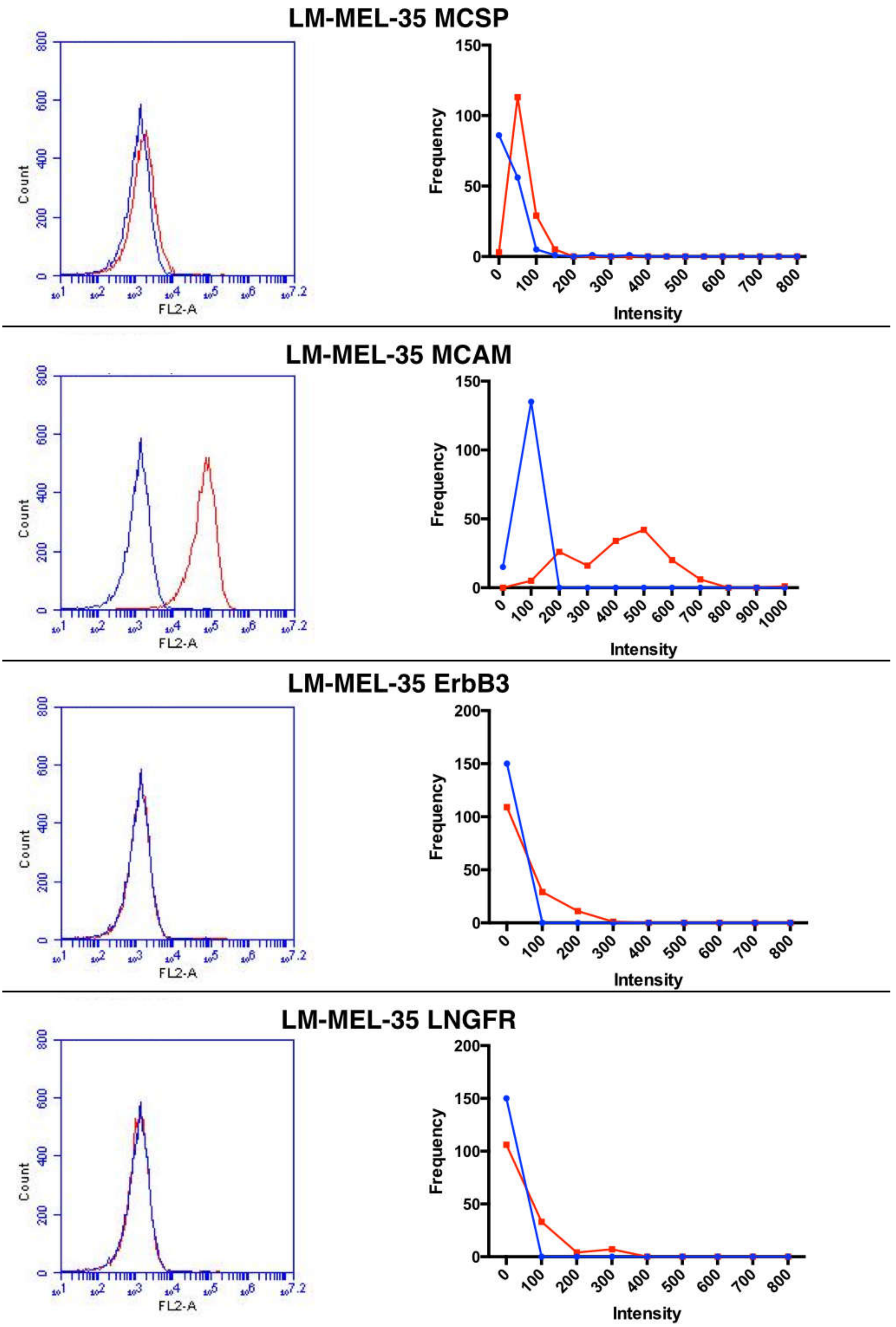

Supplementary Figure 14: Flow cytometry and SERS distribution of cell surface markers for LM-MEL-35 cell line.

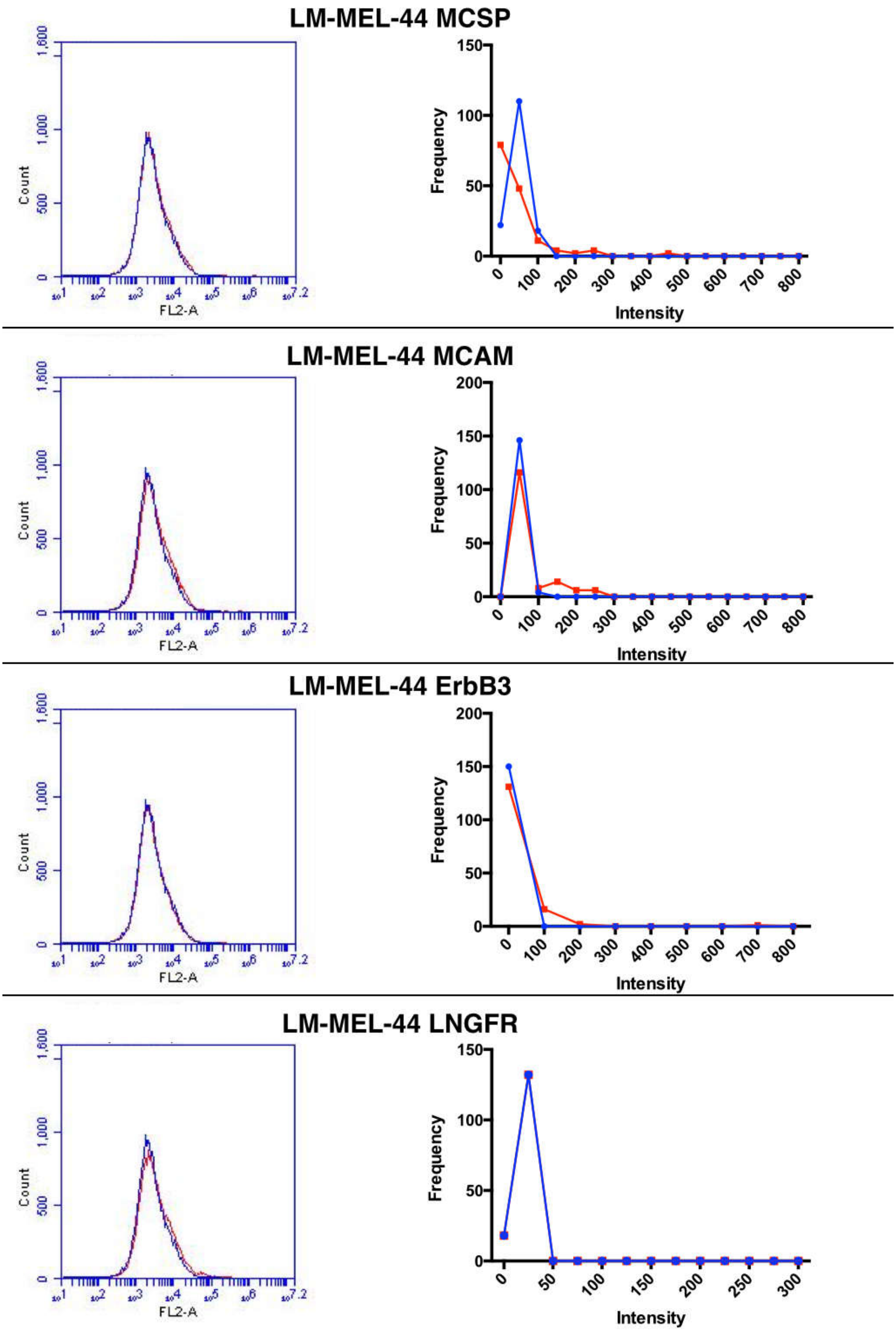

Supplementary Figure 15: Flow cytometry and SERS distribution of cell surface markers for LM-MEL-44 cell line.

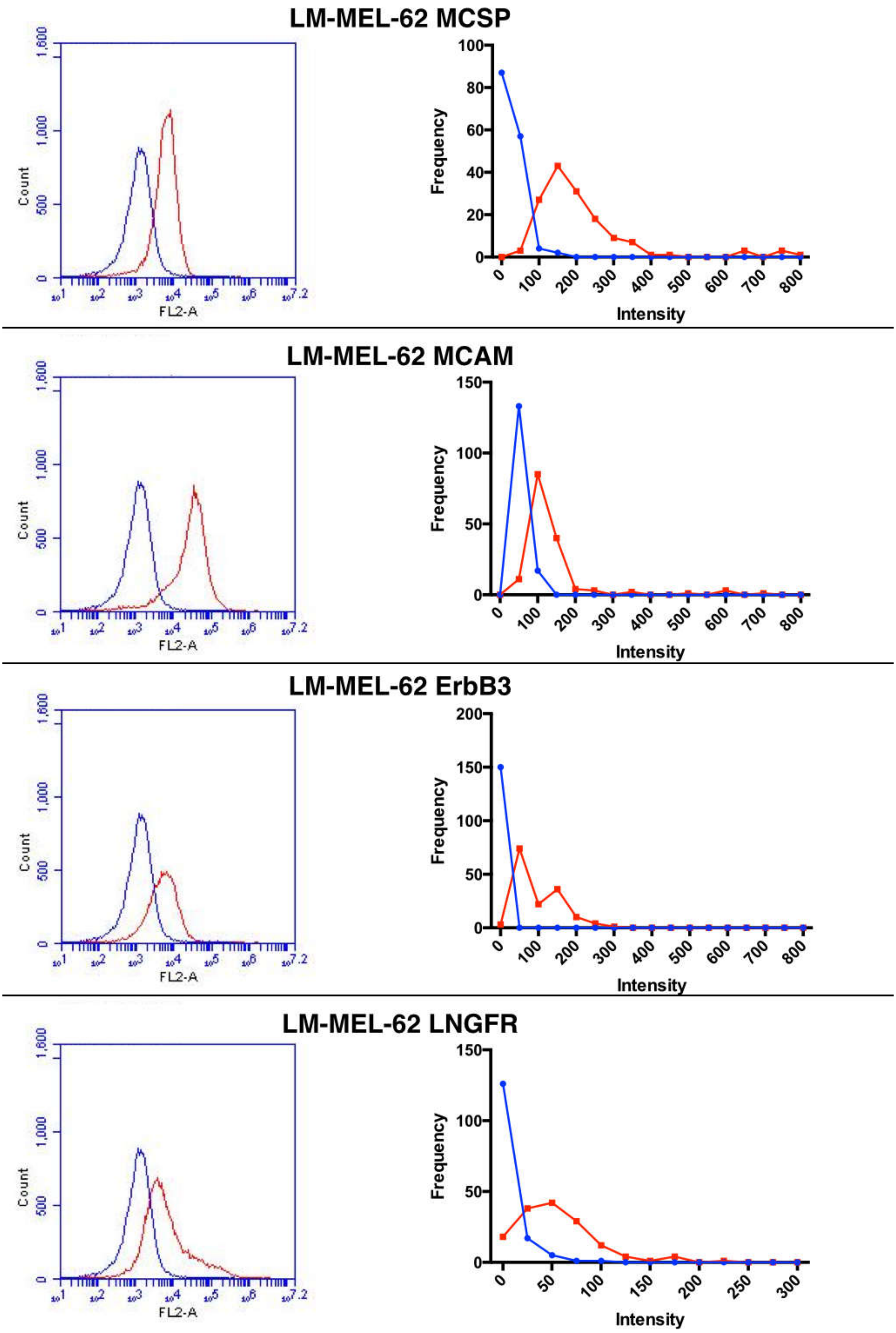

Supplementary Figure 16: Flow cytometry and Raman signal distribution of cell surface markers for LM-MEL-62 cell line.

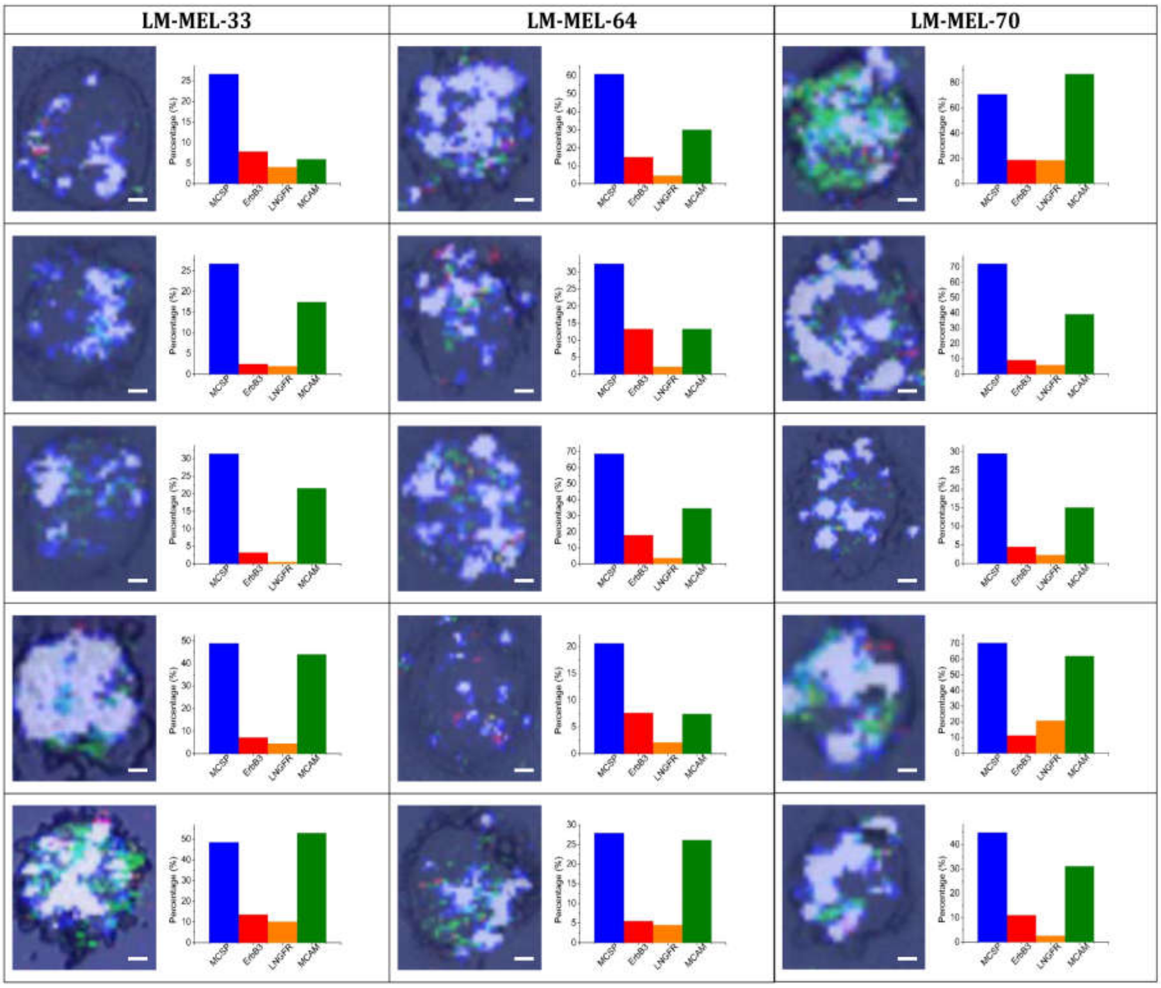

Supplementary Figure 17: Single-cell SERS images of LM-MEL-33, 64 and 70 cell lines conjugated with 4 Ab-SERS labels. Five single cells were randomly selected and imaged from each cell line. The y-axis (percentage) in the histogram is calculated by counting the numbers of dots that generated positive signals on the individual cell (i.e., Percentage = the pixel numbers of dots/the pixel numbers of per cell). Blue, red, orange, and green bars represent MCSP, ErbB3, LNGFR, and MCAM, respectively. Scale bars, 7 μm.

Before drug treatment (Day 0):

(a) LM-MEL-33 (b) LM-MEL-64 (c) LM-MEL-70

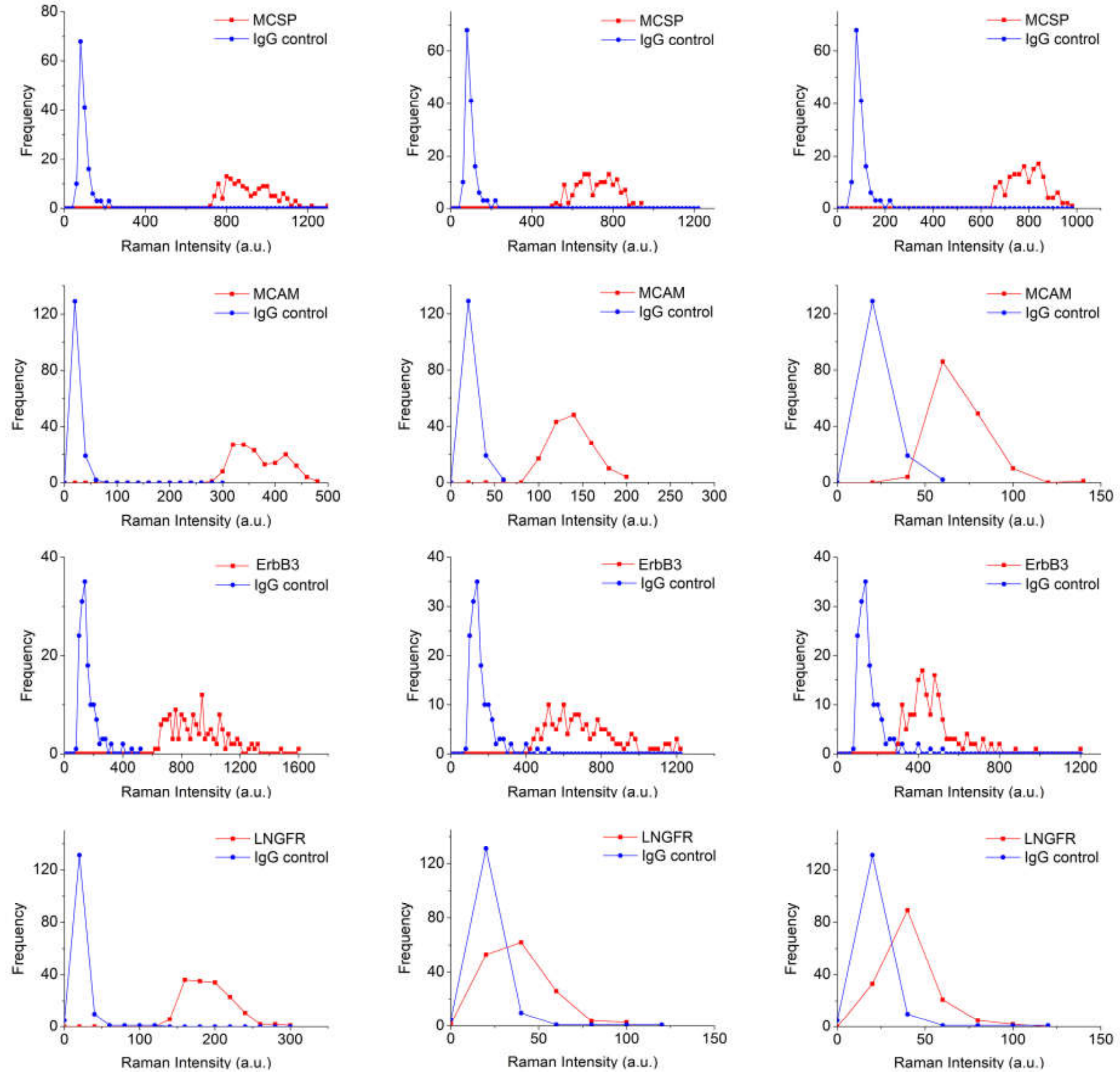

Supplementary Figure 18: SERS signal distribution for (a) LM-MEL-33, (b) LM-MEL-64, and (c) LM-MEL-70 cells before drug treatment.

On drug treatment (Day 3):

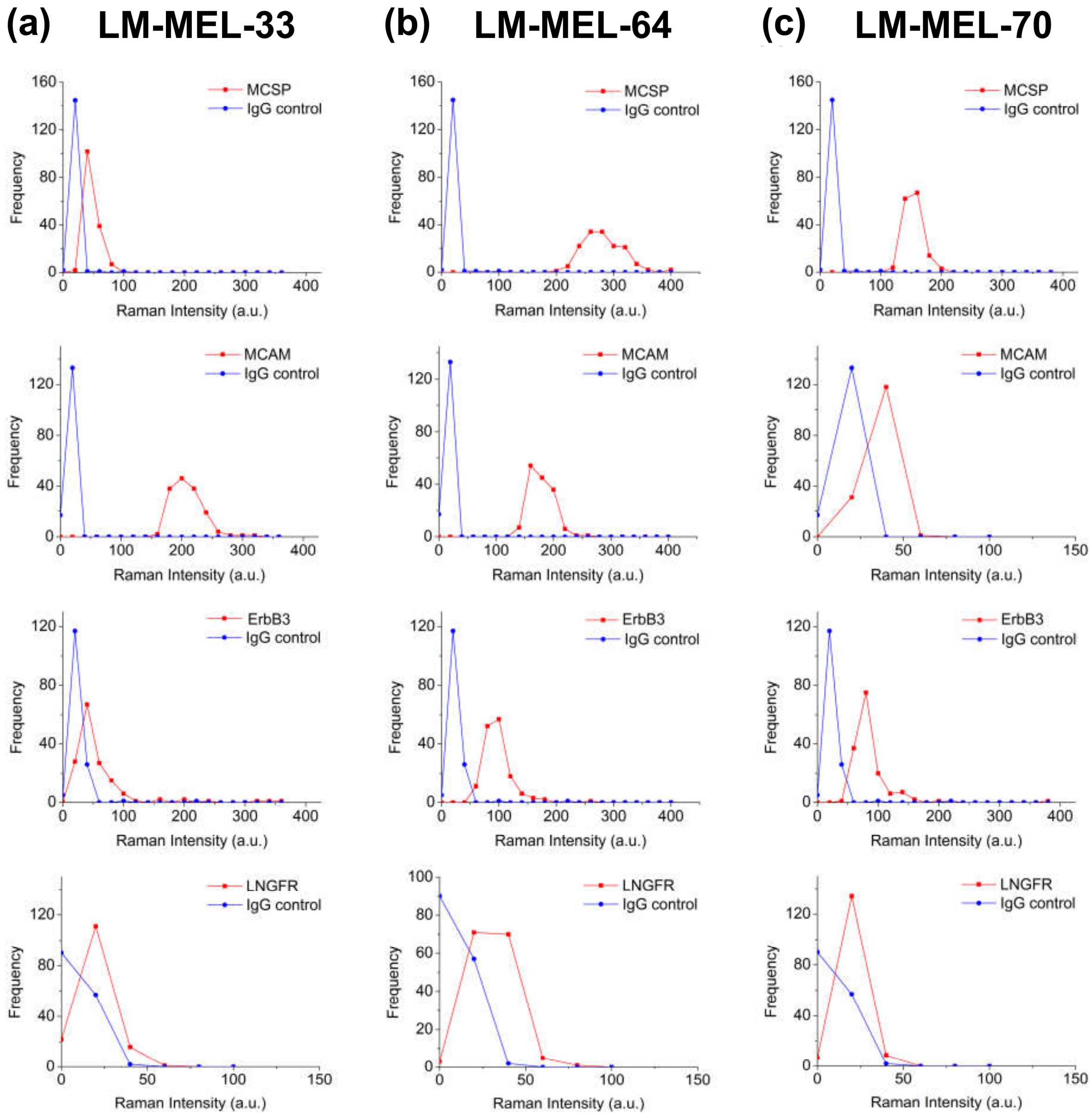

Supplementary Figure 19: SERS signal distribution for (a) LM-MEL-33, (b) LM-MEL-64, and (c) LM-MEL-70 cells having drug treatment for 3 days.

On drug treatment (Day 35):

(a) LM-MEL-33 (b) LM-MEL-64 (c) LM-MEL-70

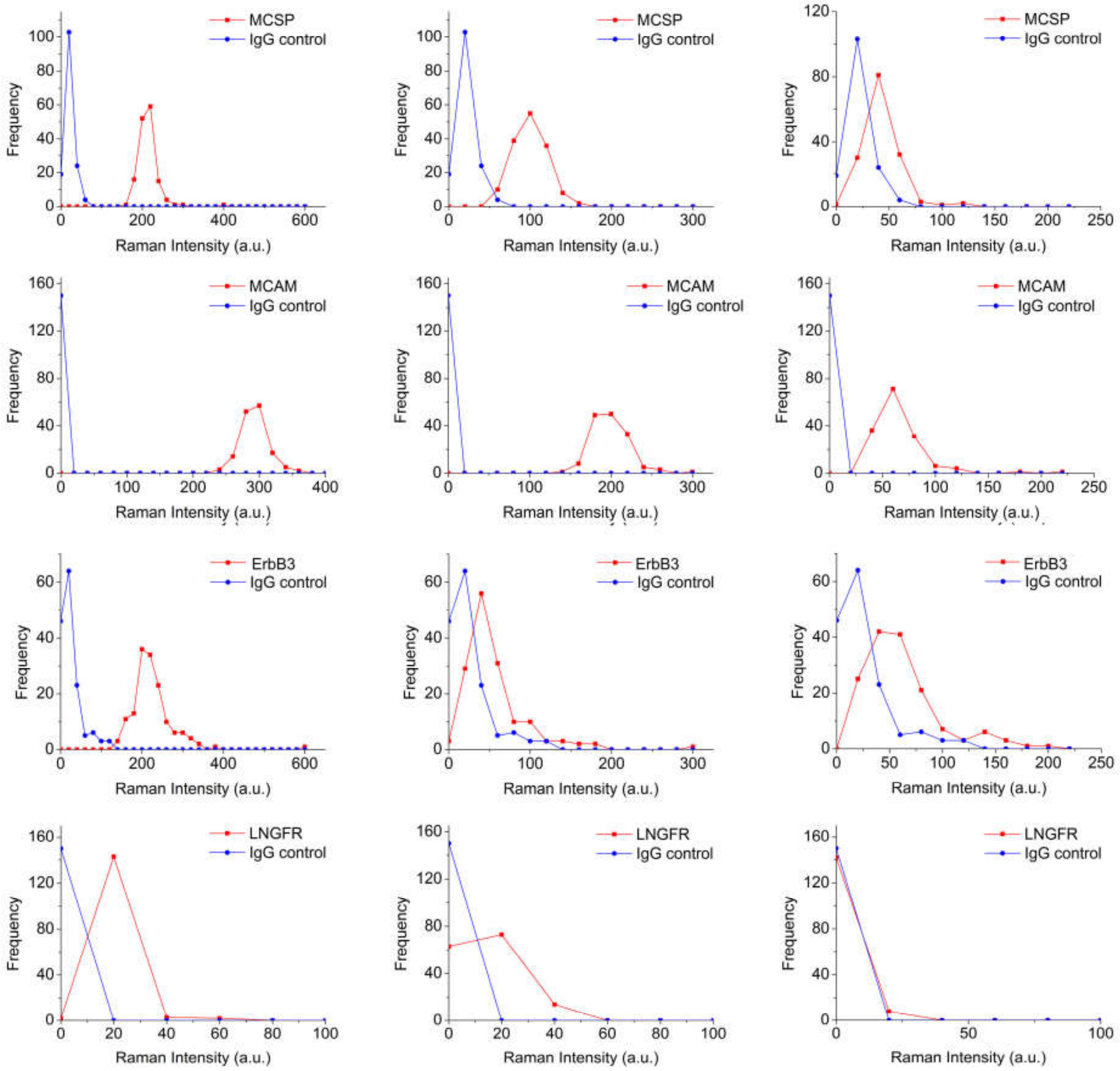

Supplementary Figure 20: SERS signal distribution for (a) LM-MEL-33, (b) LM-MEL-64, and (c) LM-MEL-70 cells having drug treatment for 35 days.

On drug treatment (Day 70):

(a) LM-MEL-33 **(b) LM-MEL-64** **(c) LM-MEL-70**

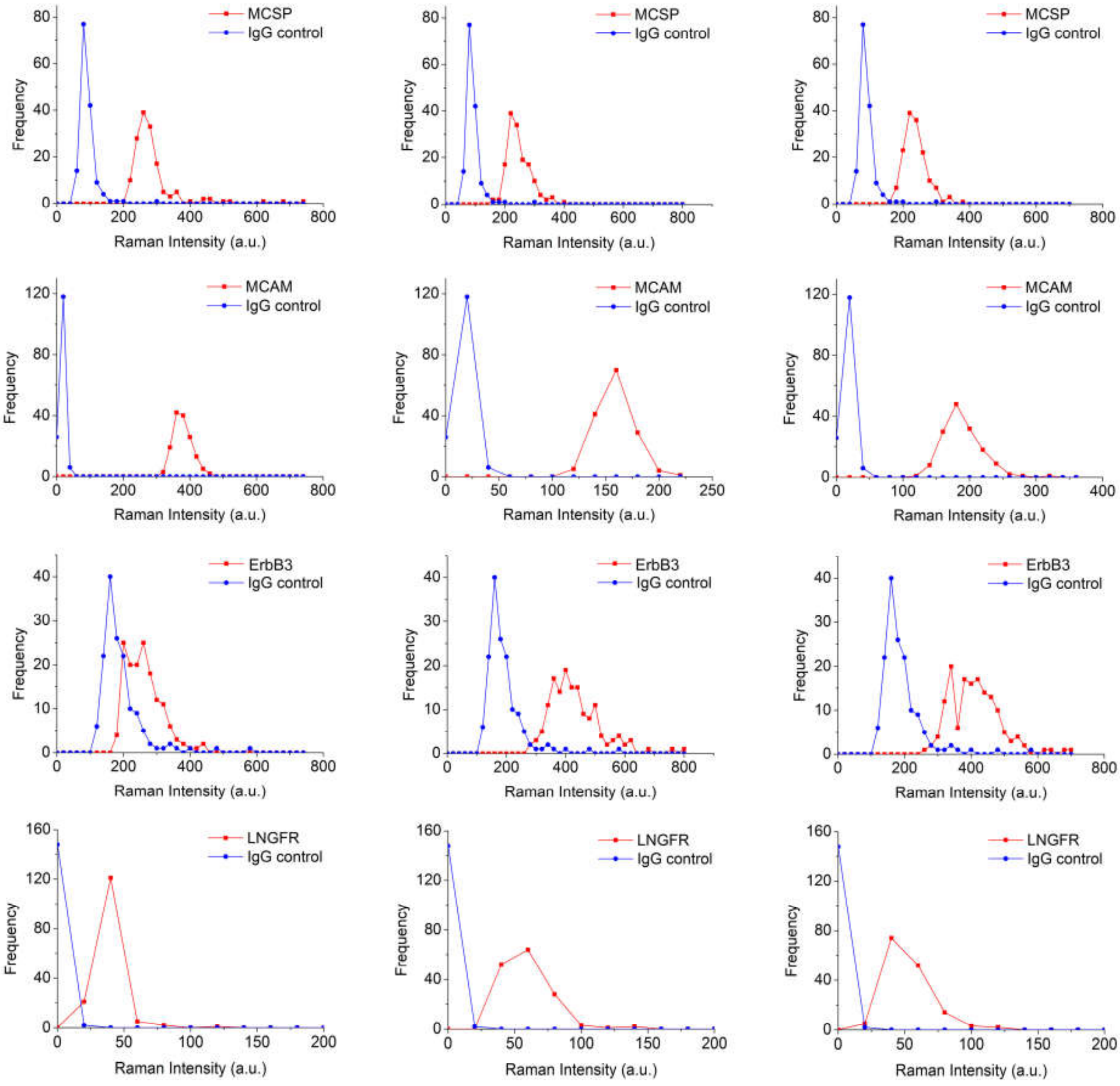

Supplementary Figure 21: SERS signal distribution for (a) LM-MEL-33, (b) LM-MEL-64, and (c) LM-MEL-70 cells having drug treatment for 70 days.

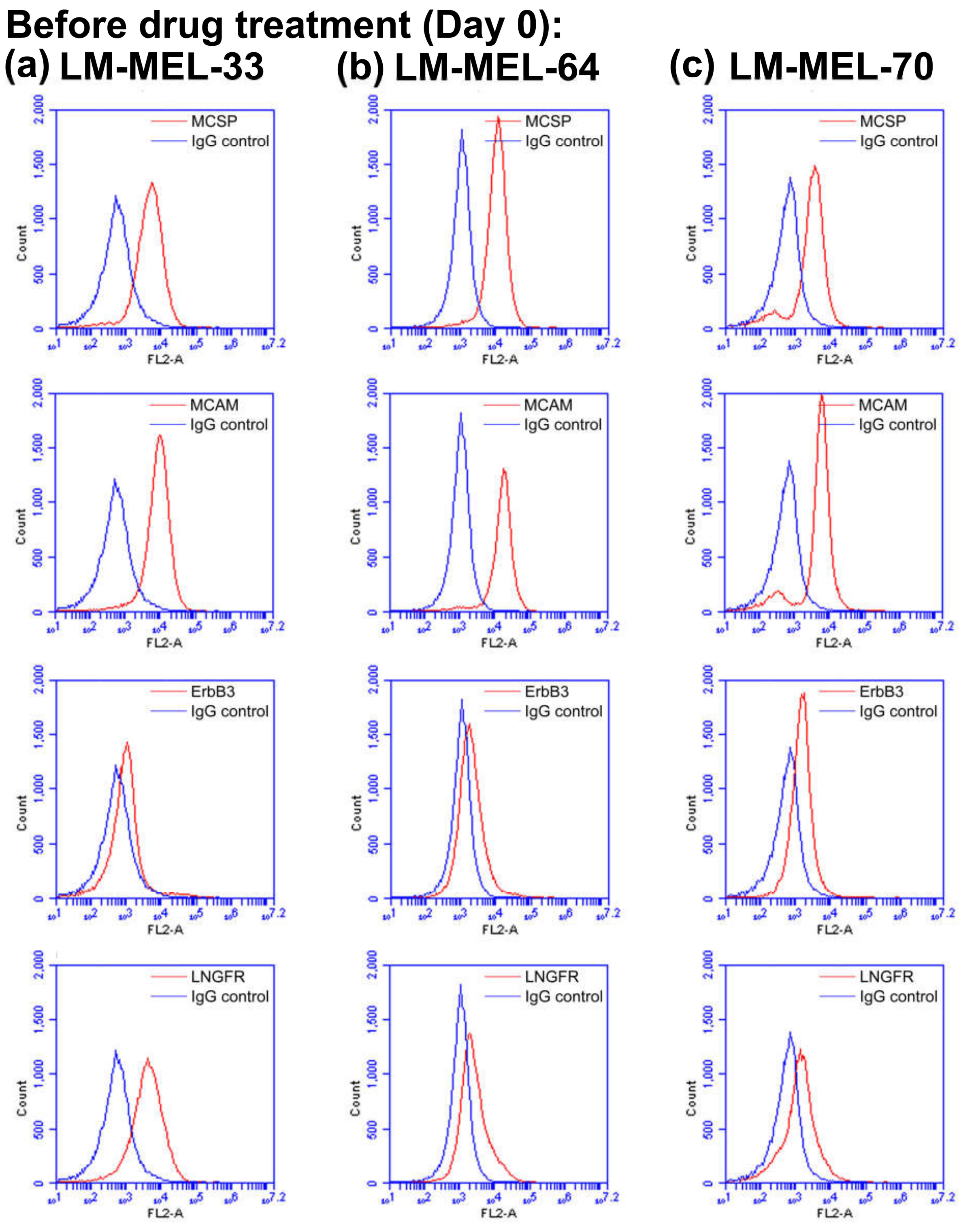

Supplementary Figure 22: Flow cytometry results for (a) LM-MEL-33, (b) LM-MEL-64, and (c) LM-MEL-70 cells before drug treatment.

On drug treatment (Day 3):

(a) LM-MEL-33 (b) LM-MEL-64 (c) LM-MEL-70

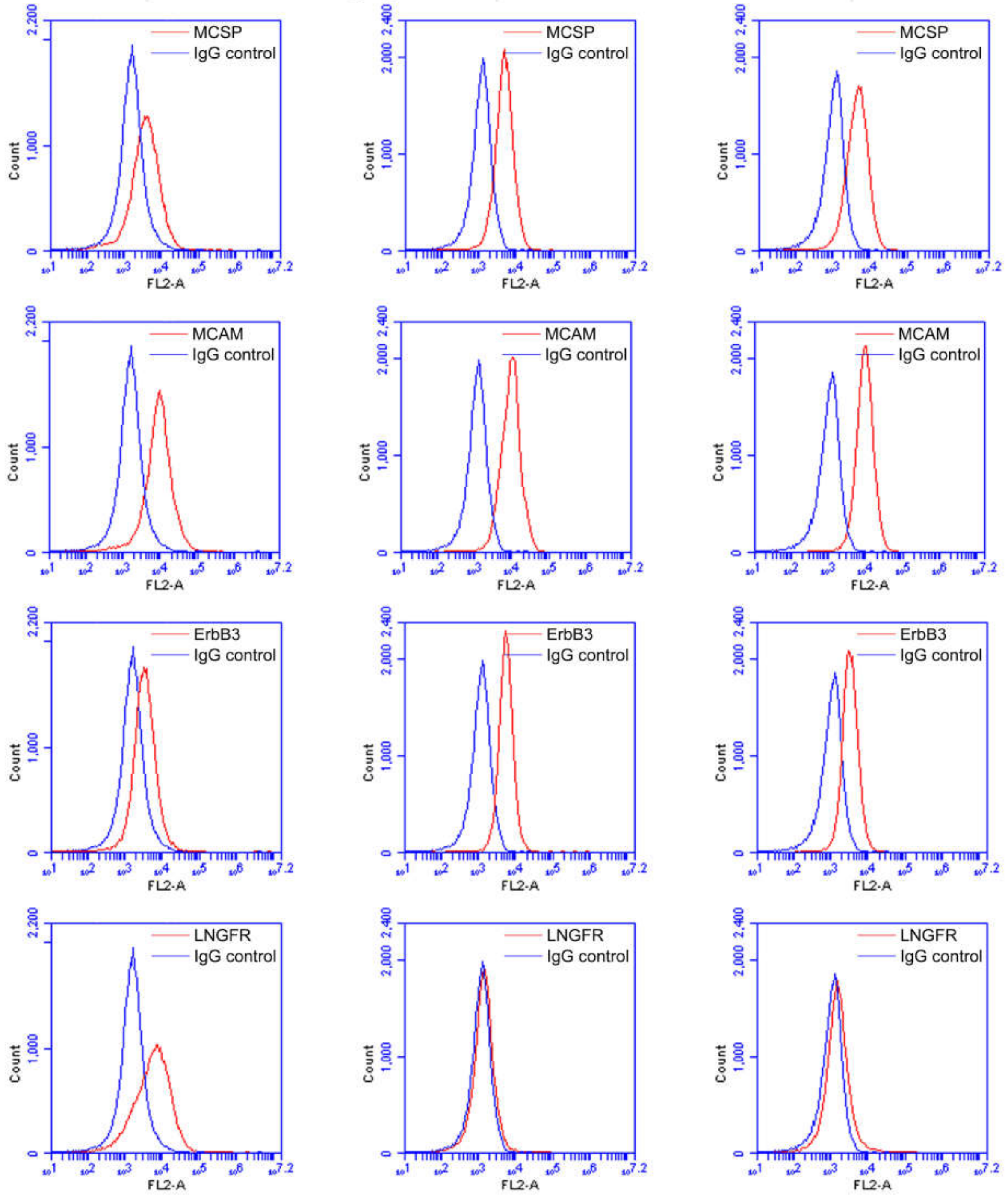

Supplementary Figure 23: Flow cytometry results for (a) LM-MEL-33, (b) LM-MEL-64, and (c) LM-MEL-70 cells having drug treatment for 3 days.

On drug treatment (Day 35):

(a) LM-MEL-33 (b) LM-MEL-64 (c) LM-MEL-70

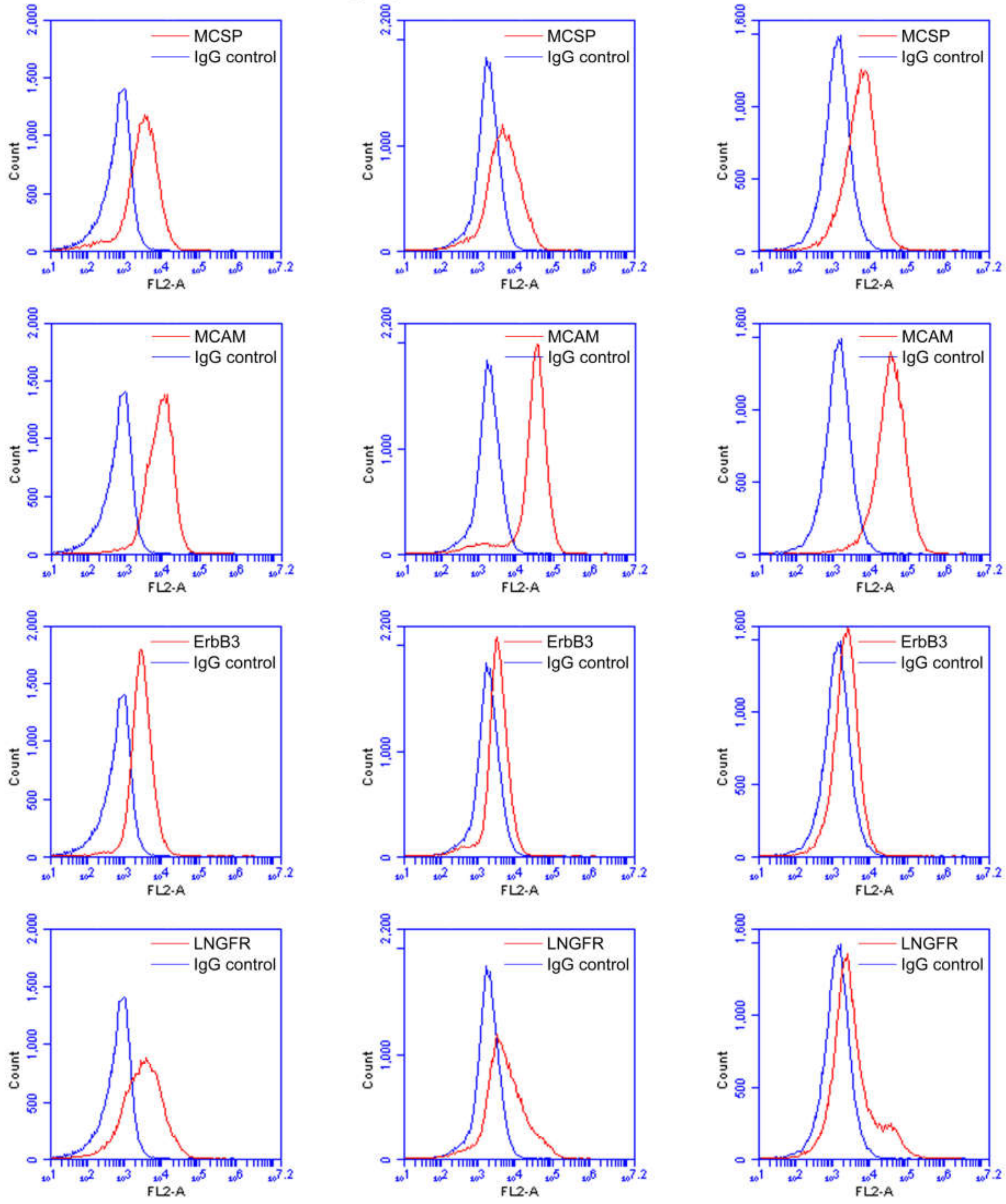

Supplementary Figure 24: Flow cytometry results for (a) LM-MEL-33, (b) LM-MEL-64, and (c) LM-MEL-70 cells having drug treatment for 35 days.

On drug treatment (Day 70):

(a) LM-MEL-33 (b) LM-MEL-64 (c) LM-MEL-70

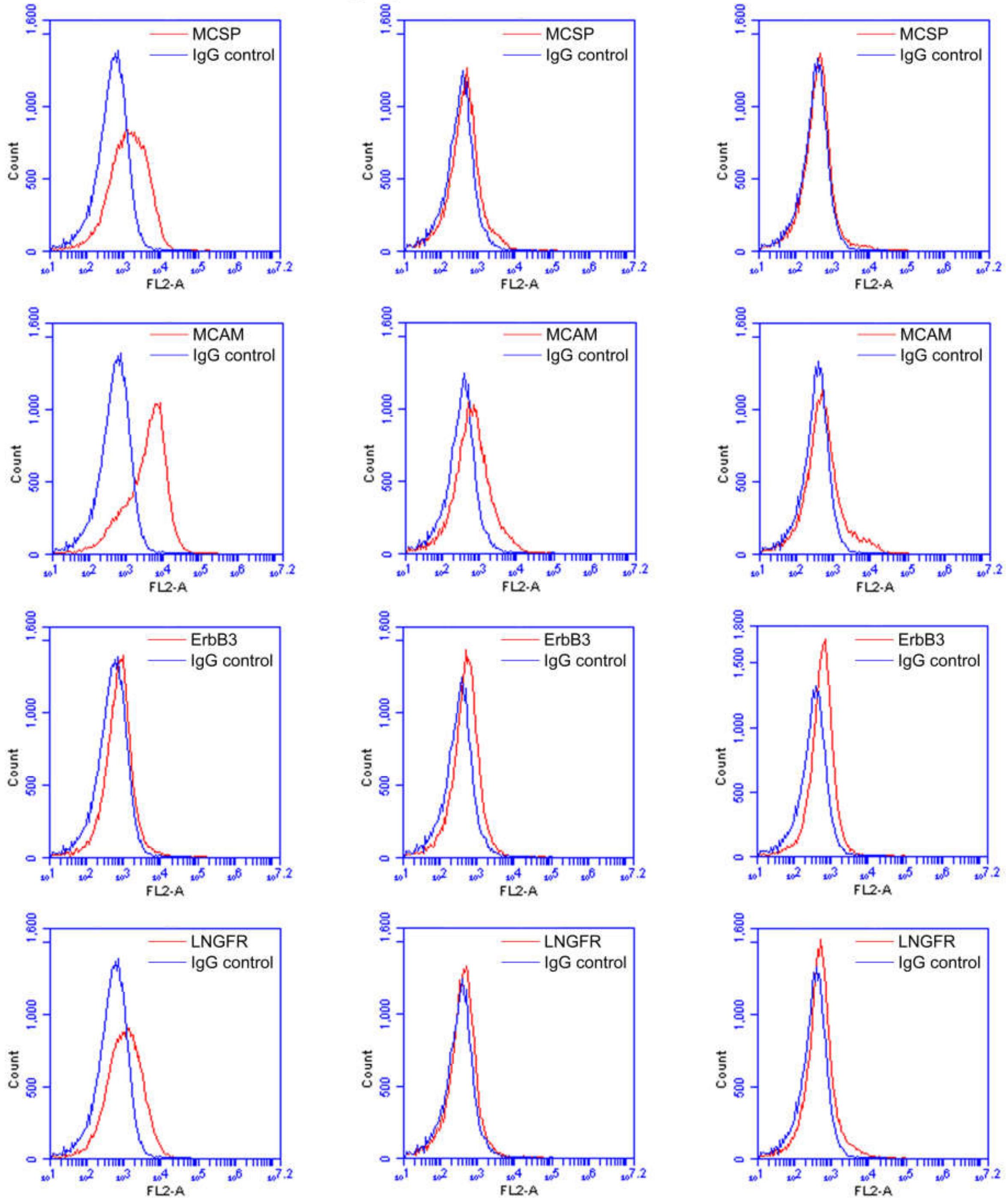

Supplementary Figure 25: Flow cytometry results (a) LM-MEL-33, (b) LM-MEL-64, and (c) LM-MEL-70 cells having drug treatment for 70 days.

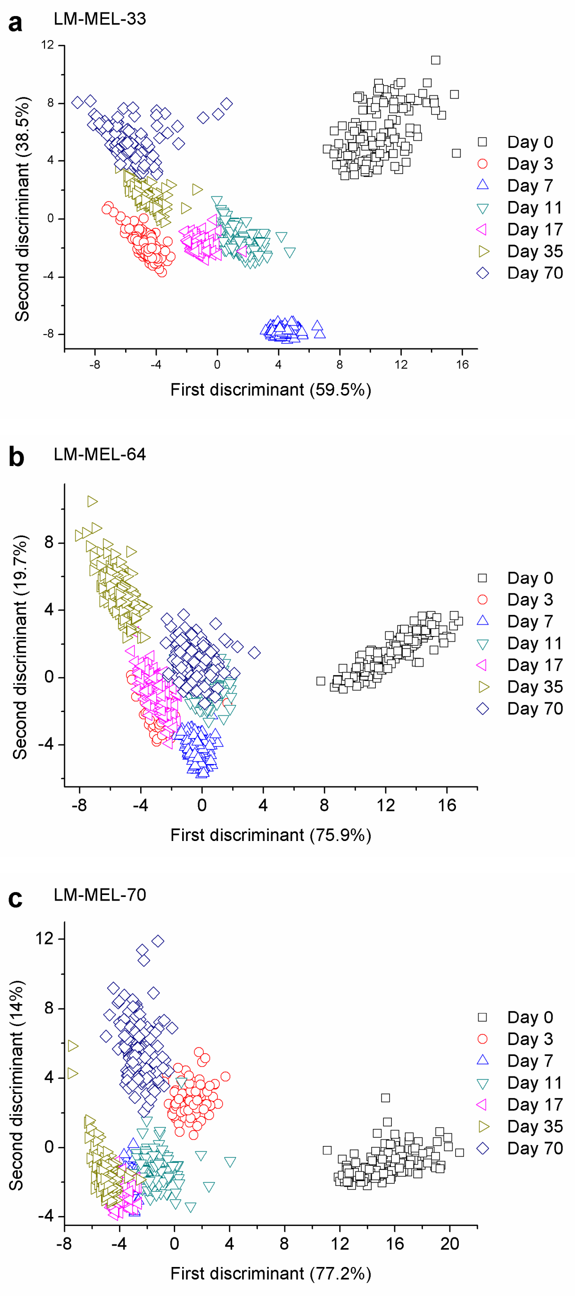

Supplementary Figure 26: Clustering of (a) LM-MEL-33, (b) LM-MEL-64, and (c) LM-MEL-70 cells in response to drug treatment after application of LDA on SERS signals.

a

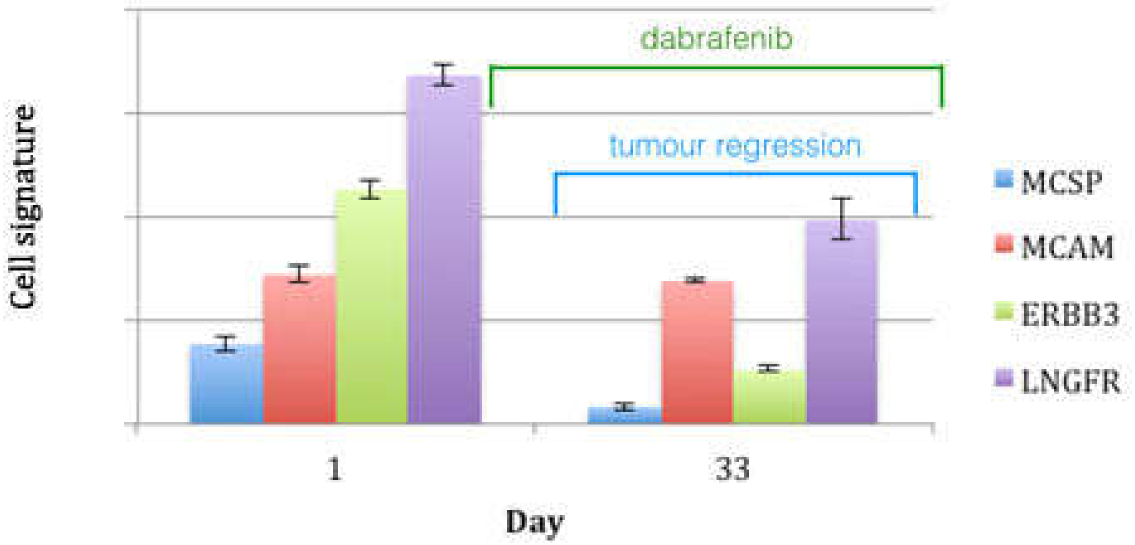
dabrafenib
tumour regression
Cell signature
MCSP
MCAM
ERBB3
LNGFR
1
33
Day

b

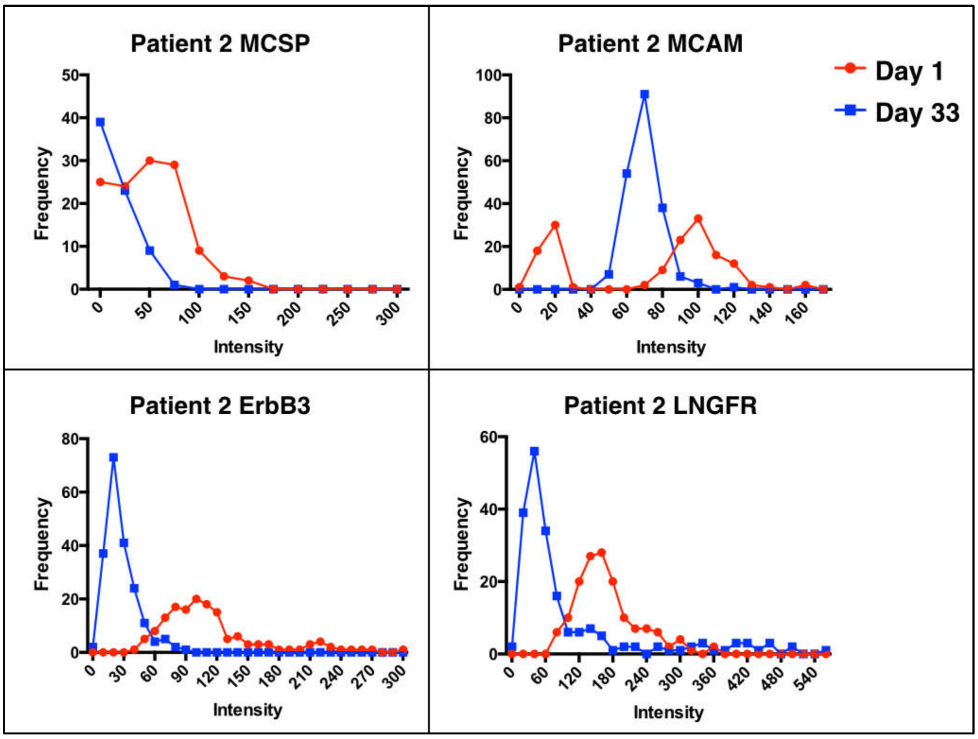
Patient 2 MCSP
Patient 2 MCAM
Patient 2 ErbB3
Patient 2 LNGFR
Day 1
Day 33
Frequency
Intensity

c

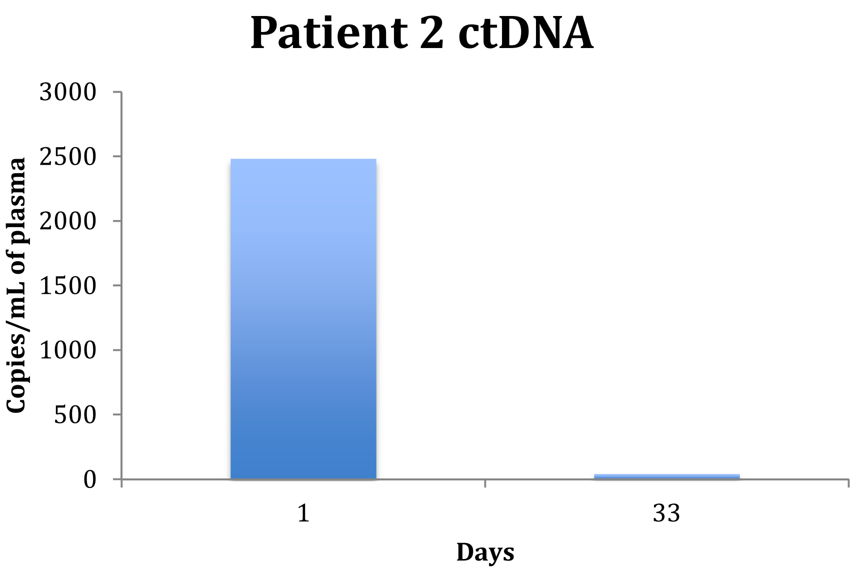

Supplementary Figure 27: CTC signatures, Raman signal distribution and circulating tumour DNA quantification[1] of patient 2.

Patient 2 presented to the hospital severely short of breath secondary to the rapidly progressive metastatic disease. The patient's tumour had previously been typed as carrying the $BRAF^{V600E}$ mutation and hence dabrafenib was started. (a) The patient's pretreatment CTC profile demonstrated detectable levels of all 4 markers with LNGFR giving the strongest signal. This profile changed following treatment with significantly lower signals in all markers except MCAM. This altered CTC signature is likely due to a combination of diminished CTC numbers and changing in population phenotype. (b) The signal distribution showed that although the average MCAM signal intensity was maintained post-treatment, the initial bi-modal signal distribution changed to a narrower single peak distribution. This signified the presence of at least 2 phenotypically different cell population pretreatment. The patient developed radiological evidence of disease progression 2 months after the second blood test. (c) Patient 2's circulating tumour DNA (ctDNA) was quantified using droplet digital PCR (ddPCR).[1] It fell significantly with successful treatment from nearly 2500 copies of $BRAF^{V600E}$ in a milliliter of plasma to just 43 copies. Data in **a** are mean ± s.d. with 150 measurements.

a

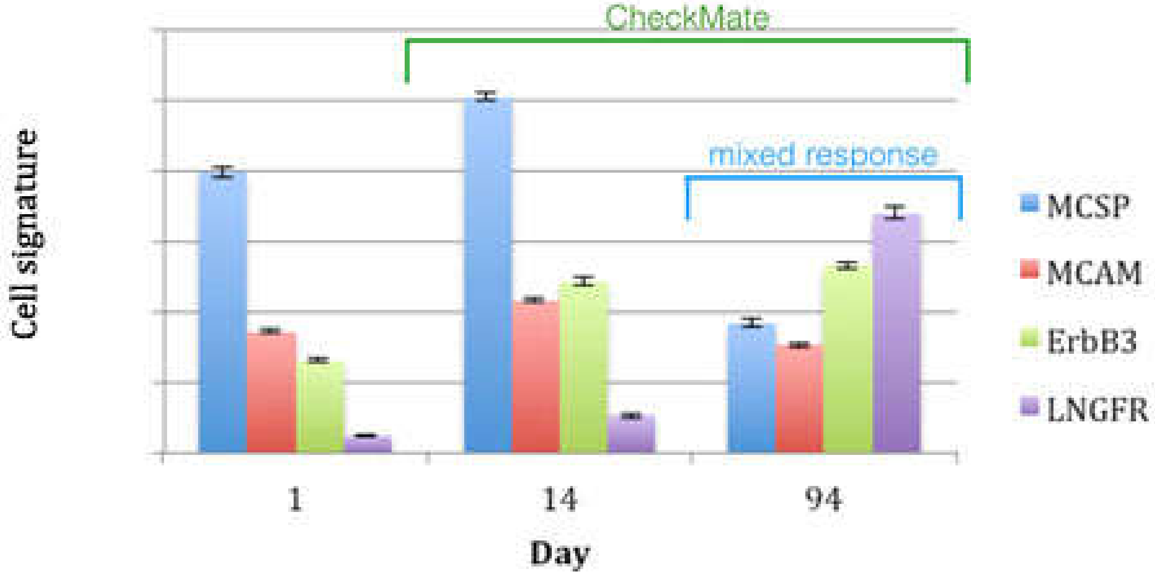

b

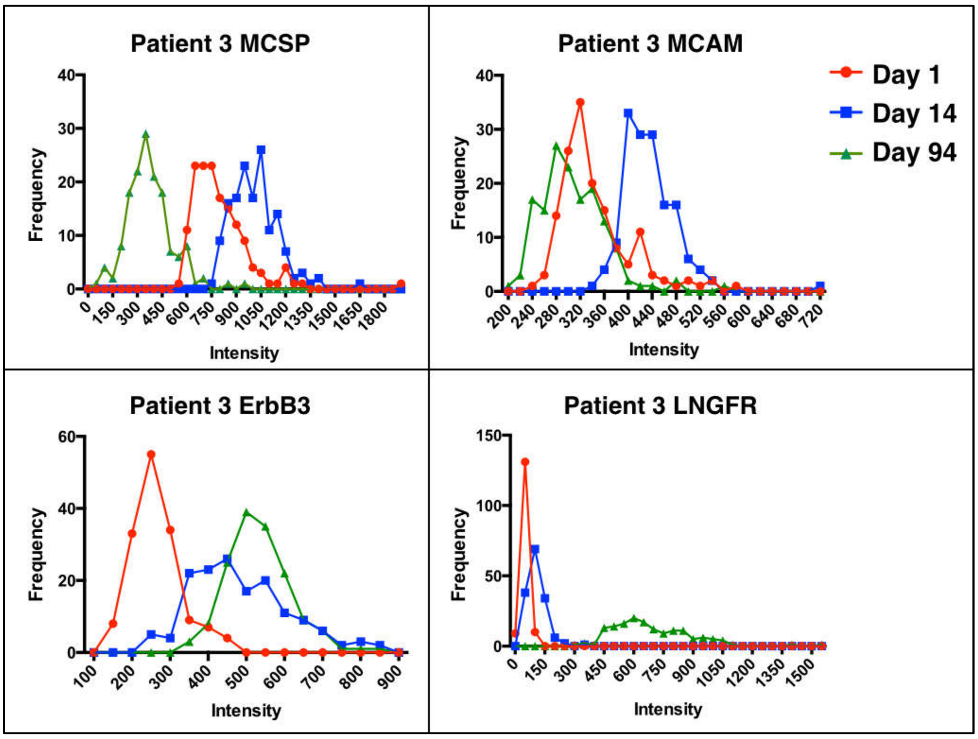

Supplementary Figure 28: CTC signatures and Raman signal distribution of patient 3.

Patient 3 was enrolled in the CheckMate trial (nivolumab +/- ipilimumab). Day 1 sample was taken just before starting the trial treatment. Repeat CT scan at day 94 showed mixed response with some tumour shrinkage and appearance of several new lesions. (a) The CTC signature is presented according to days of treatment. The signature on day 94 showed a very different picture with significantly upregulated ErbB3 and LNGFR expression but a lower MCSP expression. (b) The signal distribution showed in particularly a much higher and wider LNGFR distribution from day 94's sample. Data in **a** are mean ± s.d. with 150 measurements.

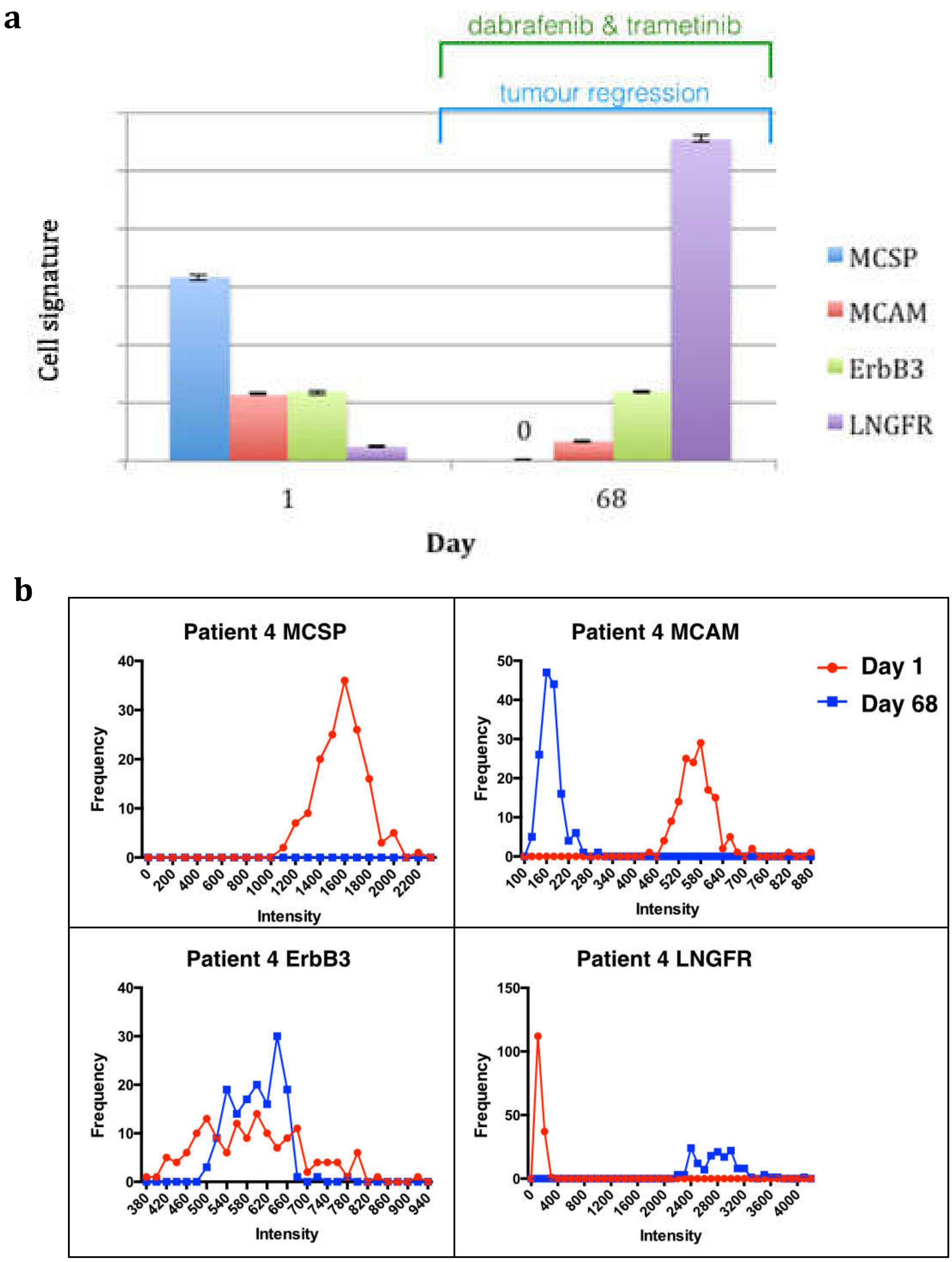

Supplementary Figure 29: CTC signatures and Raman signal distribution of patient 4.

Patient 4 was started on dabrafenib and trametinib for metastatic melanoma. (a) CTC signature showed a significant reduction in signal intensities for markers MCSP and MCAM 68 days after treatment initiation. CT scan also showed tumour regression. However, LNGFR was upregulated significantly. Patient 4 developed radiological evidence of tumour progression within 5 months after treatment initiation and was too sick for further sampling; (b) Signal distribution of the indicated markers. Data in **a** are mean ± s.d. with 150 measurements.

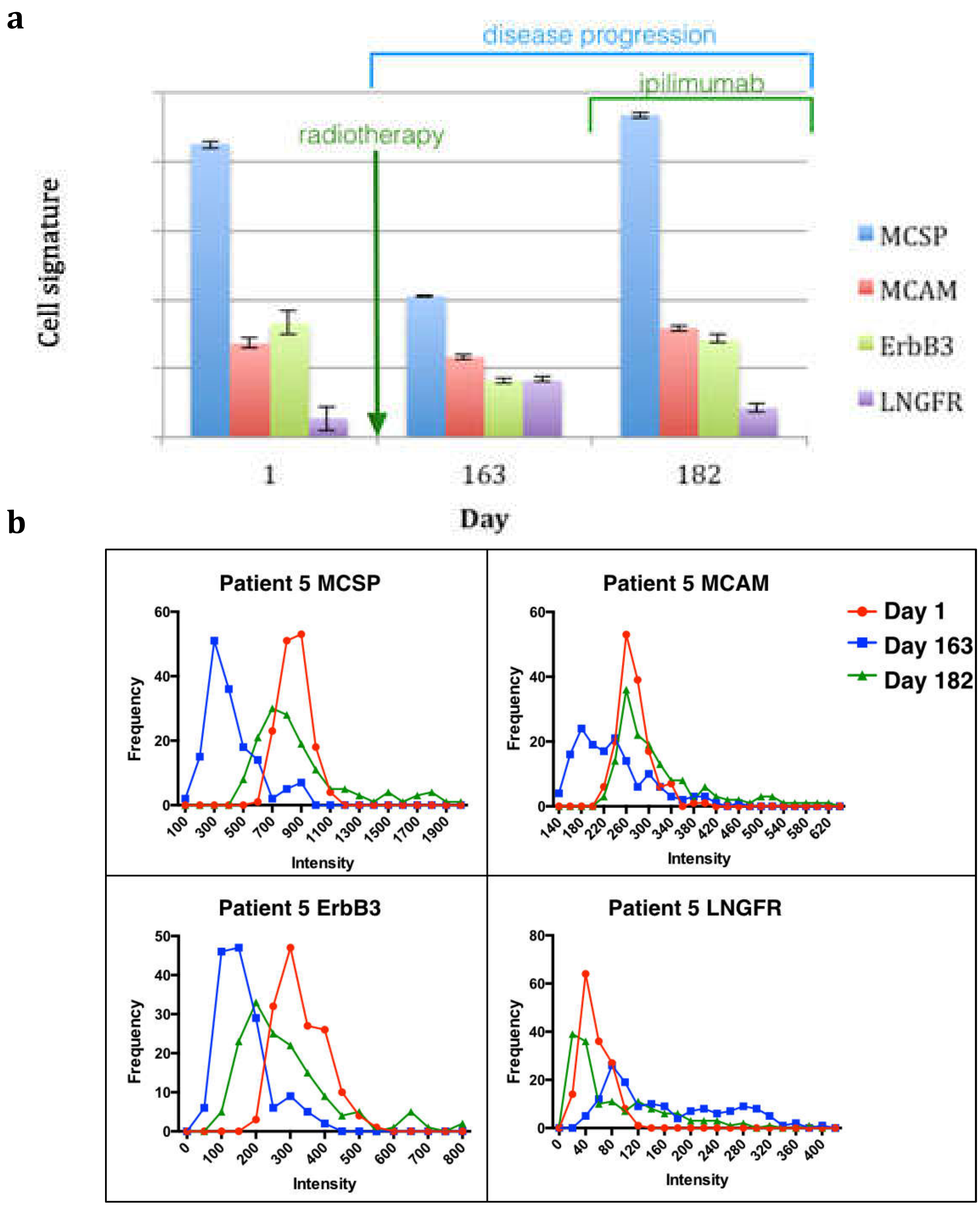

Supplementary Figure 30: CTC signatures and Raman signal distribution of patient 5.

Patient 5 received radiotherapy between day 1 and day 163. Ipilimumab was started on the day 163 when the patient developed disease progression. However, this patient deteriorated clinically and died soon after the last blood collection. (a) The CTC signature showed elevating of most signal intensities on day 182. (b) The signal distribution showed right shift (higher intensity) and widening of the distribution between the last two blood samples (blue and green). Data in **a** are mean ± s.d. with 150 measurements.

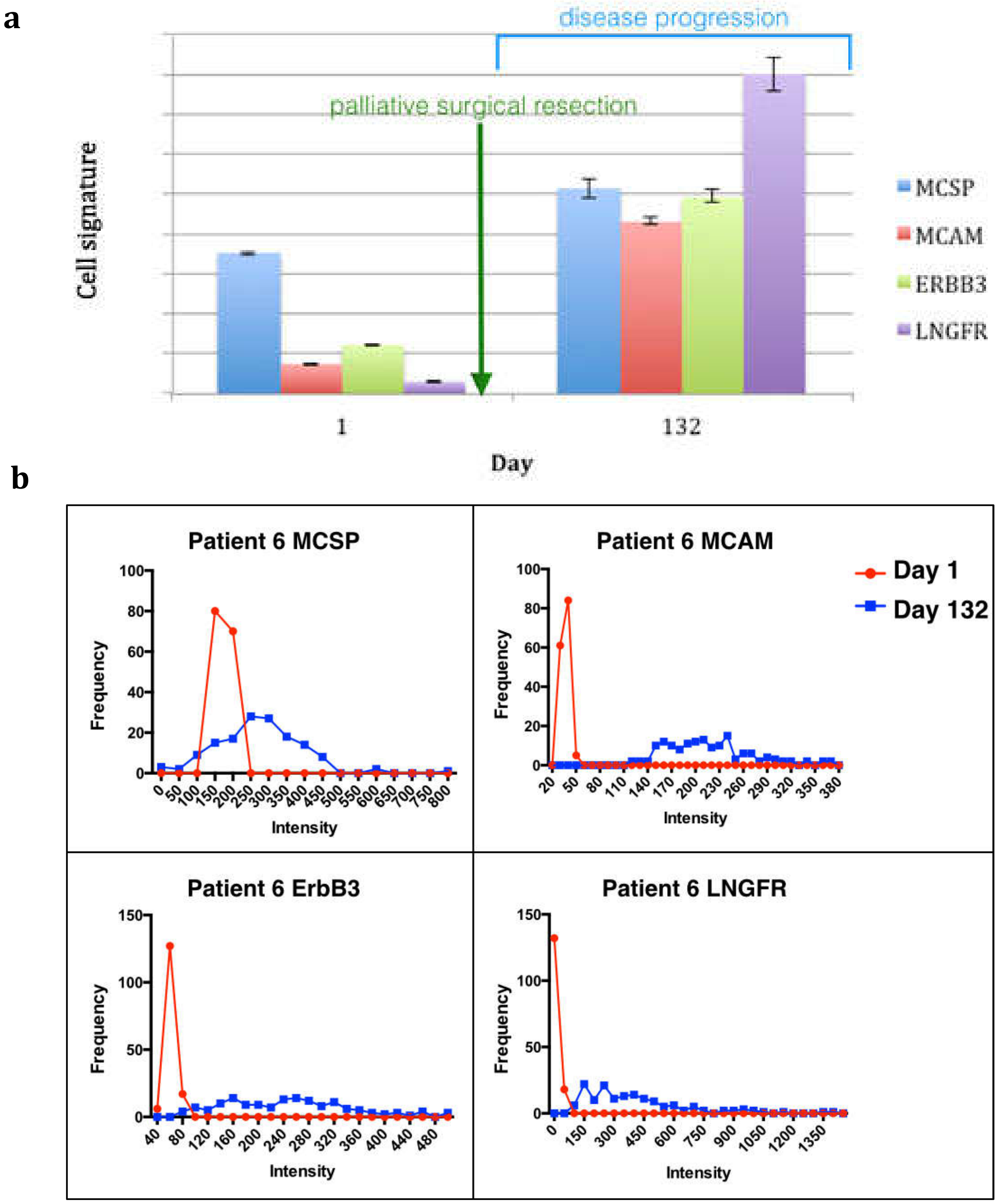

Supplementary Figure 31: CTC signatures and Raman signal distribution of patient 6.

Patient 6 first presented to the hospital with acute illness associated with metastatic melanoma. The first sample (day 1) was collected at presentation. Patient 6 received surgical resection; however, disease was too extensive and complete resection impossible. The second sample (day 132) was taken during disease progression. (a) The CTC signatures showed elevated signal intensities of all markers in the second sample. (b) The wider signal distribution in the second blood sample signified a heterogeneous CTC population. Data in **a** are mean ± s.d. with 150 measurements.

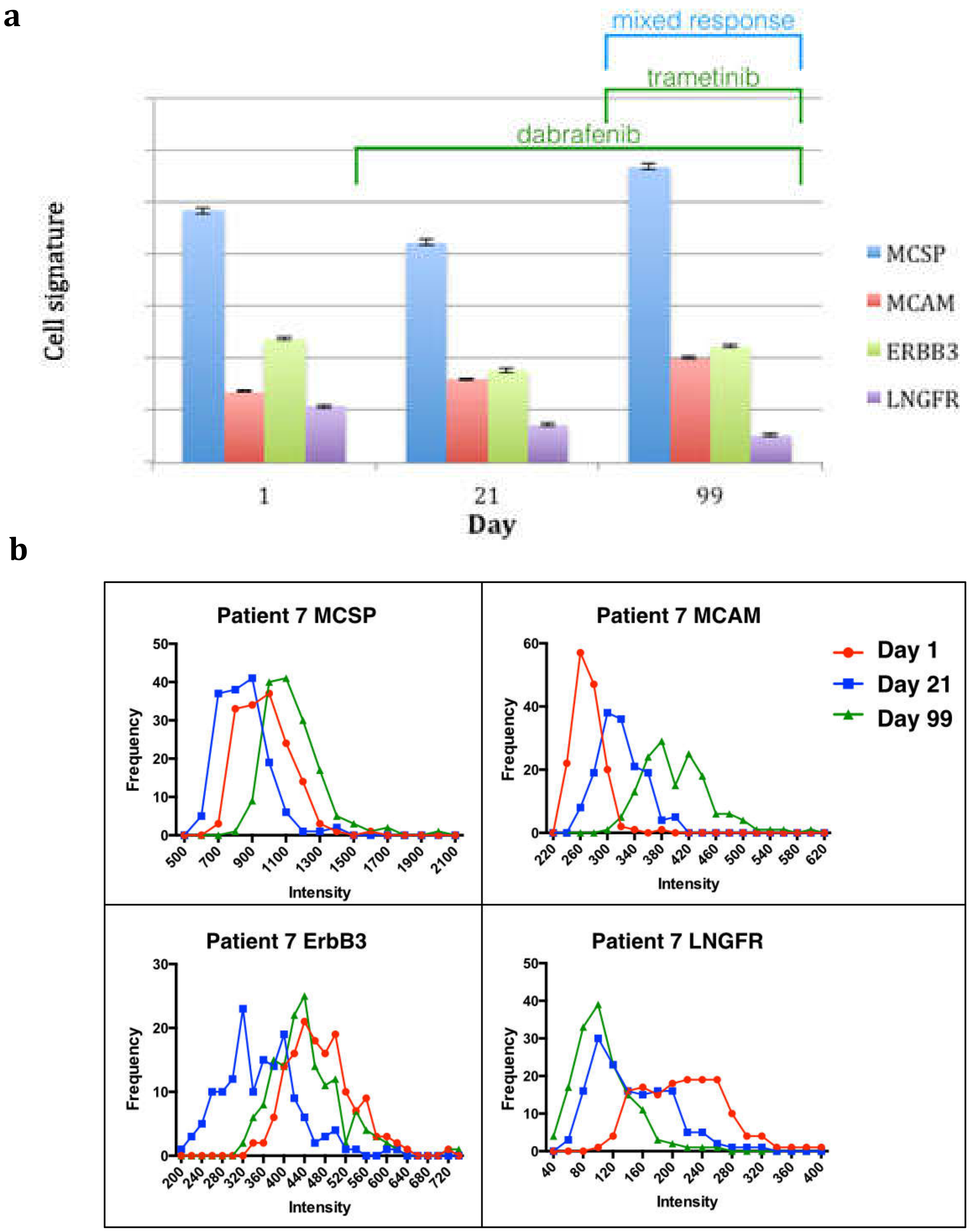

Supplementary Figure 32: CTC signatures and Raman signal distribution of patient 7.

Patient 7's first sample (day 1) was taken at diagnosis, and the patient started dabrafenib soon after. The second sample was collected 2 weeks after starting dabrafenib (day 21). On this day, trametinib was also added. The 3rd sample was taken 3 months (day 99) after the commencement of combination treatment. CT scan showed a mixed response to therapy, with some tumours shrunk but others grew. (a) The CTC signature pattern. (b) The signal distribution also showed mixed changes. Data in **a** are mean ± s.d. with 150 measurements.

a

b

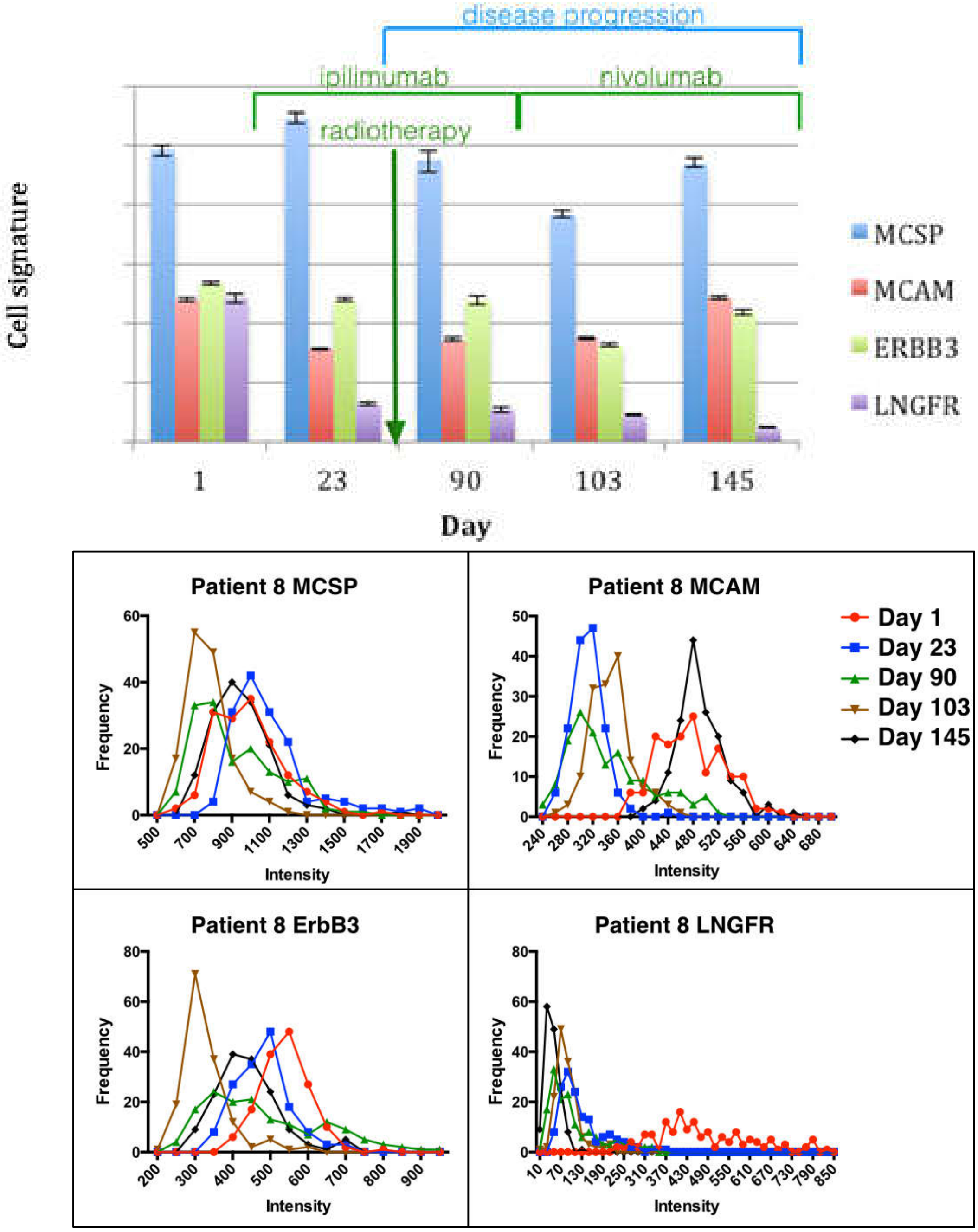

Supplementary Figure 33: CTC signatures and Raman signal distribution of patient 8.

Patient 8 started ipilimumab after the first sample collection (day 1). Patient 8 also received radiotherapy between day 23 and day 90. However, the patient did not respond to the treatment with continued disease progression on CT scan. Treatment was switched to nivolumab (a PD-1 inhibitor, which is immunotherapy). Again, the patient's disease did not respond with CT scans showing continued disease progression. (a) The CTC signature. (b) The signal distribution. Data in **a** are mean ± s.d. with 150 measurements.

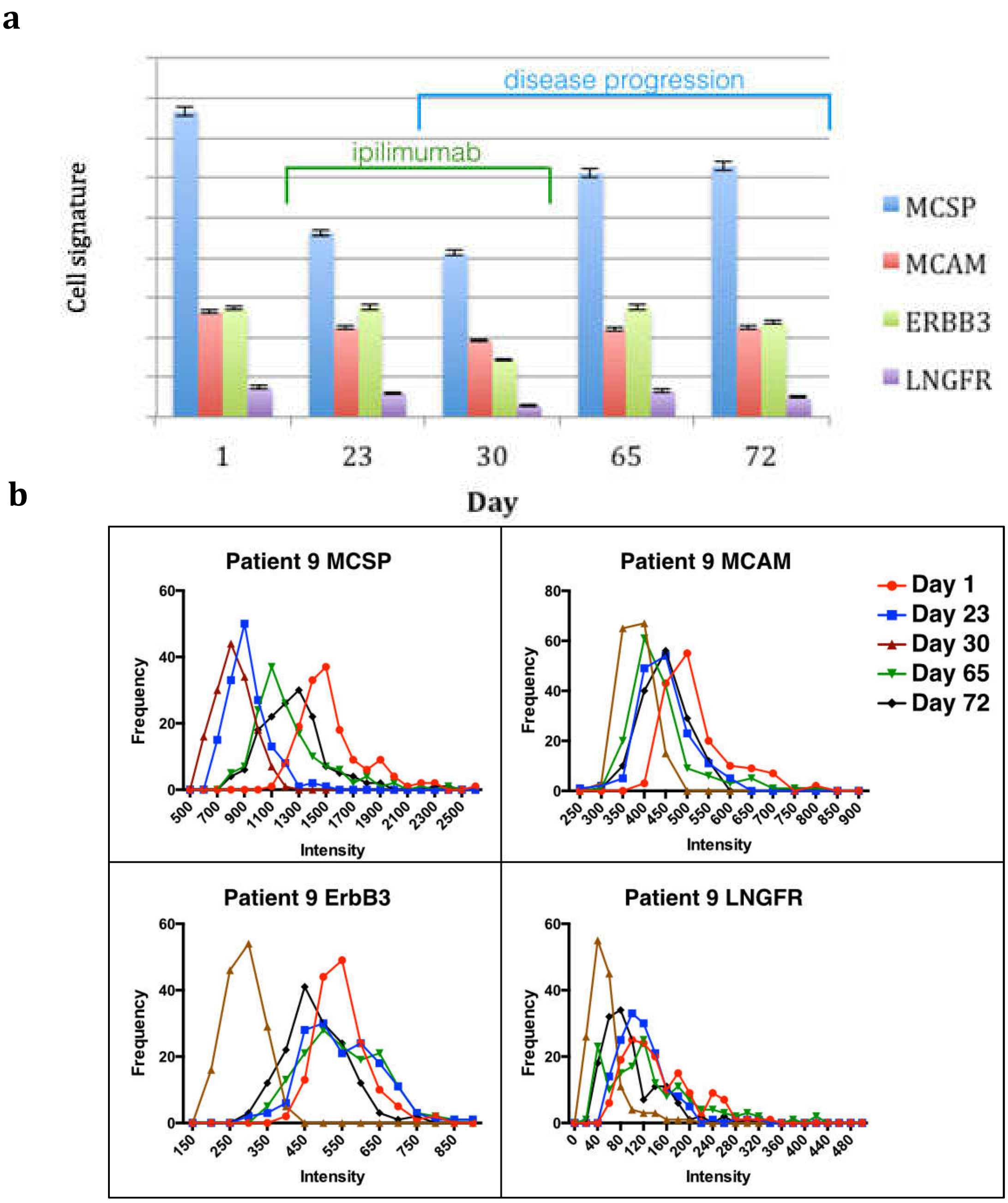

Supplementary Figure 34: CTC signatures and Raman signal distribution of patient 9.

Patient 9 started ipilimumab 1 week after the first sample was taken. However, the patient did not respond to the treatment with new lesions seen on CT at the third visit (day 30). The patient died soon after the last blood collection (day 72). (a) The CTC signature started to change on day 30. However, as the treatment stopped, it reverted to pretreatment pattern. (b) The signal distributions which also remained relatively the same. Data in **a** are mean ± s.d. with 150 measurements.

a

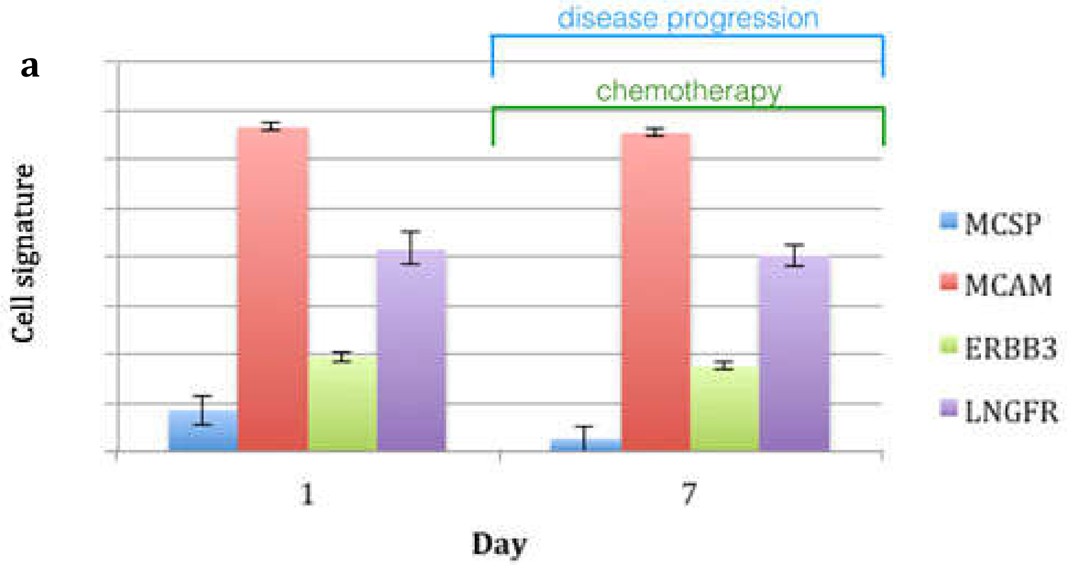
disease progression
chemotherapy
Cell signature
MCSP
MCAM
ERBB3
LNGFR
1
7
Day

b

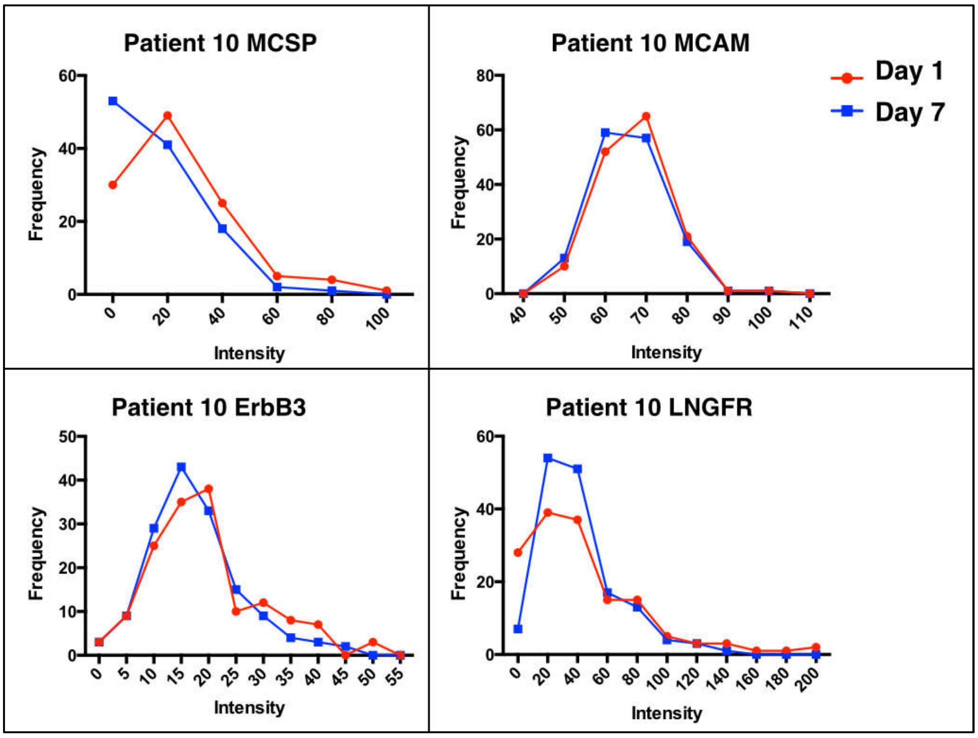
Patient 10 MCSP
Patient 10 MCAM
Patient 10 ErbB3
Patient 10 LNGFR
Day 1
Day 7
Frequency
Intensity

c

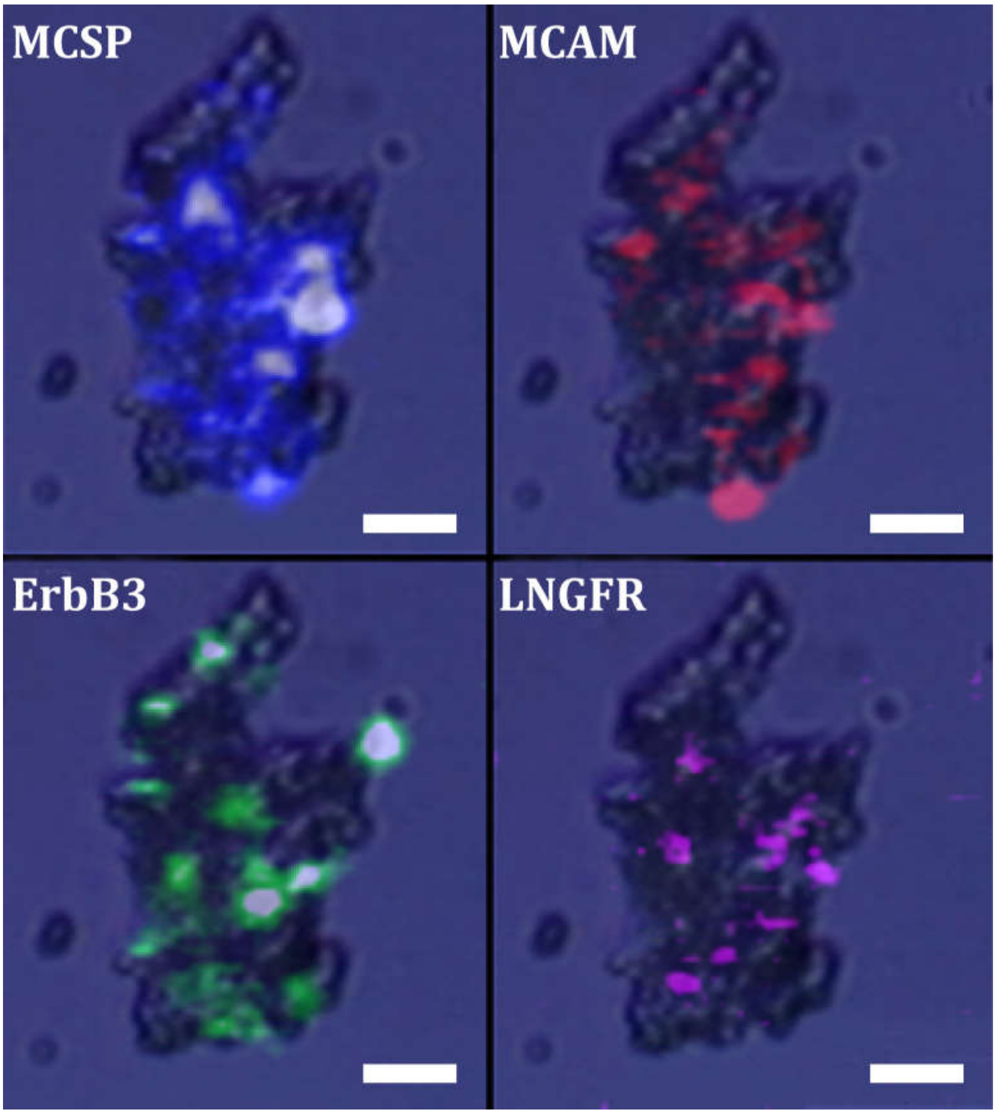

Supplementary Figure 35: CTC signatures, Raman signal distribution and SERS image of a CTC of patient 10.

Patient 10's disease had progressed while on dabrafenib and trametinib. They were ceased, and the patient was given chemotherapy as a last resort. (a) Patient 10 did not respond to continued clinical deterioration, and there was no change to the CTC signature with treatment. The patient passed away soon after the second blood test. (b) The signal distribution also remained relatively the same. (c) SERS images of the four surface marker expression as shown on the patient's CTC cluster. Data in **a** are mean ± s.d. with 150 measurements. Scale bars, 10 μm.

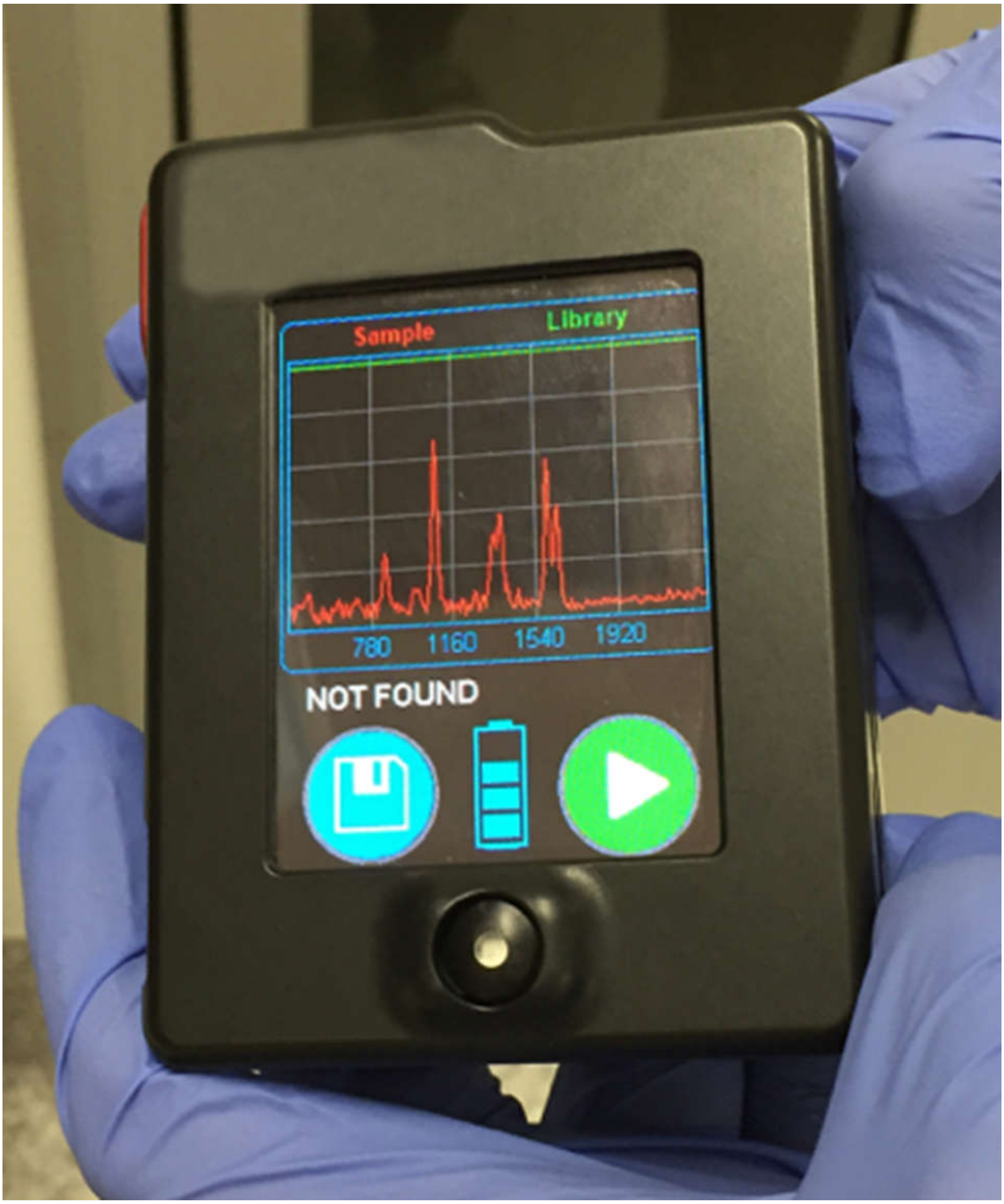

Supplementary Figure 36: A handheld Raman spectrometer with Raman spectrum from one of our patient samples.

Supplementary Table 1. Peak intensities in experimental spectra and deconvoluted models.

	Characteristic peaks (cm^{-1})	Peak intensity in experimental spectra (a.u.)	Peak intensity in deconvoluted models (a.u.)
MCSP-MBA	1075	850	850
LNGFR-MPY	1000	759	745
MCSP-MBA:LNGFR-MPY = 1:1 v/v %	1075	985	1024 (849 for MBA source)
	1000	573	577 (577 for MPY source)
MCSP-MBA:LNGFR-MPY = 1:5 v/v %	1075	424	392 (165 for MBA source)
	1000	742	728 (728 for MPY source)

Supplementary Table 2. Summary of references documenting MCSP, MCAM, ErbB3, and LNGFR expression in different cell lines and our corresponding flow cytometry data for validation.[2-9]

		Marker expression levels	
		Literatures	Experimental sMFI[§]
MCSP	SK-MEL-28	+++[*,2]	17.77
	MCF7	+[3]	1.01
MCAM	SK-MEL-28	+++[2]	45.92
	MCF7	-[4,9]	0.98
ErbB3	MCF7	++[5,6]	1.53
	SKBR3	+[5,6]	1.41
LNGFR	SK-MEL-28	++[2]	2.36
	BM-MSC	-[7,8]	1.22

*Score of reactivity of melanoma cells with antibodies: -, <5%; +, 6-20%; ++, 21-60%; and +++, 61-100%.[10]

§The specific MFI represents the ratio of the mean fluorescence intensity for the targeting marker over the mean fluorescence intensity of the isotype control (one representative experiment).

The raw flow cytometry data are shown in **Supplementary Fig. 4**.

Supplementary References

1. Tsao, S. C.-H. *et al.* Monitoring response to therapy in melanoma by quantifying circulating tumour DNA with droplet digital PCR for BRAF and NRAS mutations. *Sci. Rep.* **5,** 1–12 (2015).
2. Gray, E. S. *et al.* Circulating Melanoma Cell Subpopulations: Their Heterogeneity and Differential Responses to Treatment. *J. Invest. Dermatol.* **135**, 2040-2048 (2015).
3. Wang, X. *et al.* CSPG4 protein as a new target for the antibody-based immunotherapy of triple-negative breast cancer. *J. Natl. Cancer. Inst.* **102,** 1496–1512 (2010).
4. Zabouo, G. *et al.* CD146 expression is associated with a poor prognosis in human breast tumors and with enhanced motility in breast cancer cell lines. *Breast Cancer Res.* **11,** R1 (2009).
5. Momeny, M. *et al.* Heregulin-HER3-HER2 signaling promotes matrix metalloproteinase-dependent blood-brain-barrier transendothelial migration of human breast cancer cell lines. *Oncotarget* **6,** 3932–3946 (2015).
6. Aguilar, Z. *et al.* Biologic effects of heregulin/neu differentiation factor on normal and malignant human breast and ovarian epithelial cells. *Oncogene* **18,** 6050–6062 (1999).
7. Rojewski, M. T., Weber, B. M. & Schrezenmeier, H. Phenotypic Characterization of Mesenchymal Stem Cells from Various Tissues. *Transfus. Med. Hemother.* **35,** 168–184 (2008).
8. Soncini, M. *et al.* Isolation and characterization of mesenchymal cells from human fetal membranes. *J. Tissue. Eng. Regen. Med.* **1,** 296–305 (2007).
9. Imbert, A.-M. *et al.* CD146 expression in human breast cancer cell lines induces phenotypic and functional changes observed in Epithelial to Mesenchymal Transition. *PLoS ONE* **7,** e43752 (2012).
10. Mirkina, I. *et al.* Phenotyping of human melanoma cells reveals a unique composition of receptor targets and a subpopulation co-expressing ErbB4, EPO-R and NGF-R. *PLoS ONE* **9,** e84417 (2014).
